# Protein tyrosine phosphatase 69D is a substrate of protein O-mannosyltransferases 1-2 that is required for the wiring of sensory axons in *Drosophila*

**DOI:** 10.1016/j.jbc.2023.102890

**Published:** 2023-01-10

**Authors:** Pedro Monagas-Valentin, Robert Bridger, Ishita Chandel, Melissa Koff, Boris Novikov, Patrick Schroeder, Lance Wells, Vladislav Panin

**Affiliations:** 1 Department of Biochemistry and Biophysics, AgriLife Research, Texas A&M University, College Station, Texas, USA; 2 Complex Carbohydrate Research Center, University of Georgia, Athens, Georgia, USA

**Keywords:** protein O-mannosylation, receptor protein tyrosine phosphatase, protein O-mannosyltransferase, axon wiring, *Drosophila* sensory neurons, *Dystroglycan*, *twisted*, *rotated abdomen*, *PTP69D*, CID, collision-induced dissociation, Dg, dystroglycan, KD, knockdown, POMGnT1, protein O-mannose β1,2-N-acetylglucosaminyltransferase, POMT, protein O-mannosyltransferase, PTP69D, protein tyrosine phosphatase 69D, RPTPζ, receptor-type protein tyrosine phosphatase ζ, rt, rotated abdomen, sHCD, stepped high-energy collision dissociation, tw, twisted

## Abstract

Mutations in protein O-mannosyltransferases (POMTs) result in severe brain defects and congenital muscular dystrophies characterized by abnormal glycosylation of α-dystroglycan (α-Dg). However, neurological phenotypes of *POMT* mutants are not well understood, and the functional substrates of POMTs other than α-Dg remain unknown. Using a *Drosophila* model, here we reveal that Dg alone cannot account for the phenotypes of *POMT* mutants, and identify *Protein tyrosine phosphatase 69D* (*PTP69D*) as a gene interacting with *POMTs* in producing the abdomen rotation phenotype. Using RNAi-mediated knockdown, mutant alleles, and a dominant-negative form of PTP69D, we reveal that *PTP69D* is required for the wiring of larval sensory axons. We also found that *PTP69D* and *POMT* genes interact in this process, and that their interactions lead to complex synergistic or antagonistic effects on axon wiring phenotypes, depending on the mode of genetic manipulation. Using glycoproteomic approaches, we further characterized the glycosylation of the PTP69D transgenic construct expressed in genetic strains with different levels of POMT activity. We found that the PTP69D construct carries many O-linked mannose modifications when expressed in *Drosophila* with wild-type or ectopically upregulated expression of POMTs. These modifications were absent in *POMT* mutants, suggesting that PTP69D is a substrate of POMT-mediated O-mannosylation. Taken together, our results indicate that PTP69D is a novel functional substrate of POMTs that is required for axon connectivity. This mechanism of POMT-mediated regulation of receptor-type protein tyrosine phosphatase functions could potentially be conserved in mammals and may shed new light on the etiology of neurological defects in muscular dystrophies.

Post-translational modification of proteins with glycans are essential regulators of biological processes in normal and pathological conditions, affecting a broad range of molecular and cellular interactions in all organisms (1). Glycosylation plays key roles in neural development and physiology, whereas defects in glycosylation can cause severe neurological abnormalities (2, 3, 4, 5). Protein O-mannosylation is among the glycosylation pathways known to be crucial for neural and neuromuscular development and physiology (6, 7, 8). Several O-mannosylation enzymes modify serine and threonine residues of mammalian proteins with O-linked mannose, including POMT1–2 (**p**rotein **O**-**m**annosyl**t**ransferases 1–2), TMTC1-4 (**t**rans**m**embrane and **t**etratricopeptide repeat–**c**ontaining proteins 1–4, also known as **t**ransmembrane O-**m**annosyltransferases **t**argeting **c**adherins), and TMEM260 (**t**rans**mem**brane protein 260) (8, 9, 10, 11, 12). The substrate specificities of these enzymes are not well understood, but they are thought to represent distinct families of glycosyltransferases that modify nonoverlapping subsets of protein targets (8).

*POMT1–2* are highly conserved in evolution and present as a distinct family of two genes in a wide range of animal species, from *Drosophila* to humans (6, 13, 14, 15, 16). POMT1 and POMT2 function together as an enzymatic complex that modifies and supports the activity of dystroglycan (Dg), a membrane protein that serves as a link between the extracellular matrix and cytoskeleton (11, 12, 17). POMTs collaborate with several other glycosylation enzymes to synthesize specialized O-mannosyl glycans on the extracellular part of α-Dg, including matriglycan, a long glycan structure that is required for α-Dg binding to its extracellular ligands (17). Genetic defects affecting this glycan cause severe congenital muscular dystrophies termed dystroglycanopathies (7, 17, 18, 19, 20).

Dystroglycanopathies are commonly associated with neurological phenotypes, ranging from severe brain malformation, such as cobblestone lissencephaly, to mental retardation without obvious structural brain abnormalities (21, 22, 23, 24, 25). Although substantial progress has been achieved in understanding the function of POMTs in muscles, the roles of these enzymes in the nervous system and the pathogenic mechanisms underlying neurological phenotypes of POMT mutants are not well understood (26). Furthermore, the relationship between Dg, the only well-studied molecular target of POMTs, and the POMT-mediated functions in the nervous system remain to be elucidated. Recent studies identified several novel POMT-dependent substrates of protein O-mannosylation in mammalian cells; however, the functional importance of their O-mannosylation is not known (9, 27). Intriguingly, O-mannosyl glycans are known to be present on receptor-type protein tyrosine phosphatase ζ (RPTPζ) that is highly expressed in the brain and intimately involved in neural development (28, 29, 30, 31, 32). However, RPTPζ remains an orphan substrate of O-mannosylation as the enzymes that modify it with O-mannose remain unknown. RPTP proteins compose a large family of evolutionarily conserved signaling receptors that regulate function of their substrates by reversing the effect of tyrosine kinases (33). RPTPs show overall similar structure of type I transmembrane proteins commonly including several fibronectin type 3 repeats and immunoglobulin-like domains within their extracellular region and one to two protein tyrosine phosphatase domains in the cytoplasmic part (34, 35). They play essential functions in the nervous system, regulating axon guidance and regeneration, synapse formation, and plasticity (33, 36). The function of O-mannosylation on RPTPζ is not well understood, and whether this modification is also present on other RPTPs and evolutionarily conserved is not known.

In our previous studies, we found that the function of *POMT* genes is conserved in *Drosophila*. *POMT1* and *POMT2*, also known in *Drosophila* as *rotated abdomen* (*rt*) and *twisted* (*tw*), respectively, function together to modify Dg with O-linked mannose (37). Mutations in *POMT1* and *POMT2* result in identical phenotypes, including a conspicuous misalignment of abdominal segments causing a twisted appearance of the abdomen in adult flies, the so-called, “abdomen rotation” phenotype (15, 16, 38, 39). More recently, we found that a similar phenotype of body torsion is also present in *POMT* mutant embryos, which was caused by abnormal muscle contractions and defects in wiring of sensory axons (40). Axons of class IV dendritic arborization neurons terminate in the ventral ganglion in a stereotypic ladder pattern made of commissural and longitudinal branches (41). This pattern is affected by *POMT* mutations that cause thickening of commissural connections and defects in branching of the sensory axons (40). Notably, mutations in *Dg* do not cause the body torsion phenotype, suggesting that this phenotype in *POMT* mutants is associated with the effect on other functionally important targets of POMTs in the nervous system (40).

In the current study, we investigated the functional relationship between *POMTs* and *Protein tyrosine posphatase 69D* (*PTP69D*), a gene encoding an RPTP that is known to be involved in axon guidance of motoneurons and the development of axon connectivity in the visual system and the giant fiber circuitry (34, 42, 43, 44, 45). We found that *PTP69D* is also required for proper wiring of sensory axons in the ventral ganglion of *Drosophila* larvae. Our experiments uncovered strong genetic interactions between *PTP69D* and *POMTs* in establishing axon connections of sensory neurons. Furthermore, we revealed that the extracellular domain of PTP69D is O-mannosylated *in vivo* in a *POMT*-dependent manner. Together, our results demonstrated that PTP69D is a novel functional target of POMTs in the nervous system, and that PTP69D and POMTs collaborate in wiring axon connections of class IV sensory neurons.

## Results

### Dg is not the main functional target of POMTs in producing the abdomen rotation phenotype of *POMT* mutants

**Figure 1:**
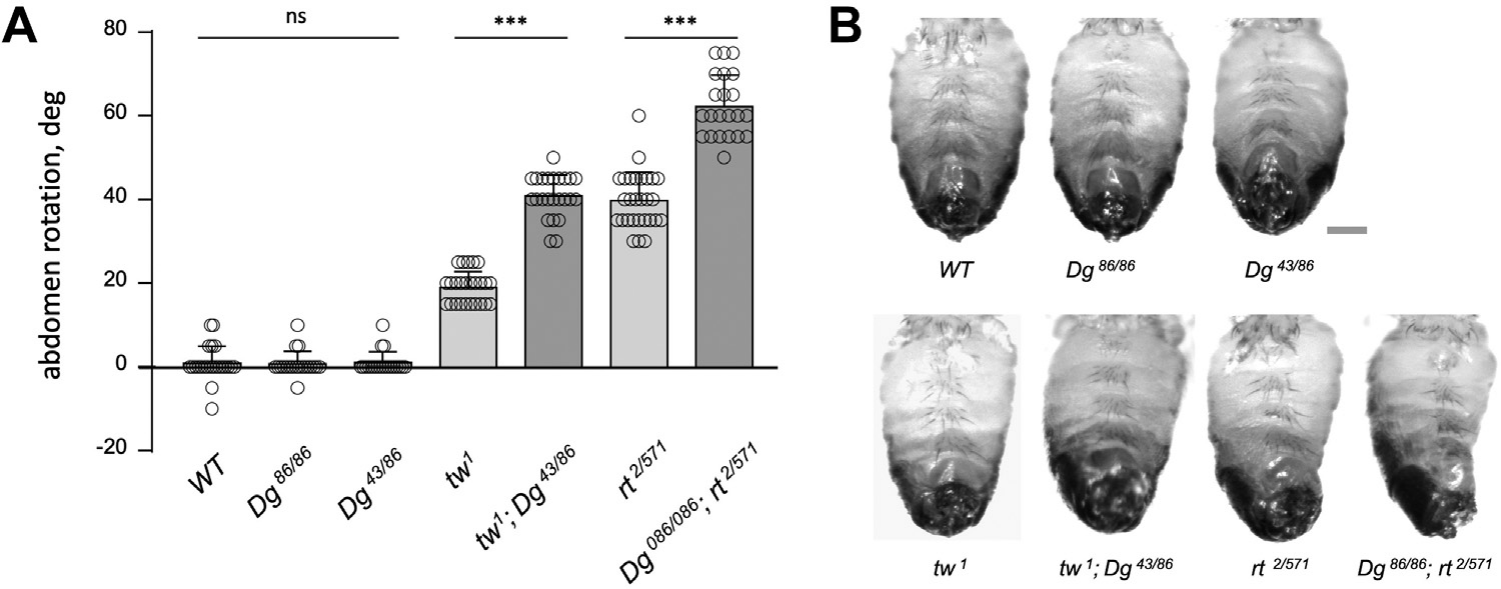
**Genetic interactions between *POMTs* (*rt and tw*) and *Dg*.***A*, *Dg* homozygous null mutants (*Dg*^*86/86*^ and *Dg*^*43/86*^) do not show the abdomen rotation phenotype characteristic for *rt* and *tw* mutants. The abdomen rotation is prominent in *rt-Dg* and *tw-Dg* double mutants indicating that *Dg* is not epistatic to *POMTs*, and thus other targets of POMTs are predicted to be involved in generating the phenotype. Positive and negative values denote clockwise counterclockwise rotation, respectively. Error bars are SEMs. Statistical significance was analyzed by one-way ANOVA with post hoc Tukey HSD test. The level of statistical significance: ns, not significant, ∗∗∗, *p* < 0.001. At least 20 males were analyzed for each genotype. *B*, representative examples of the abdomen rotation phenotype of *Drosophila* adult males with *rt, tw*, and *Dg* mutations. Only abdomens are shown. Ventral view, anterior is up. The scale bar represents 300 μm. Dg, dystroglycan; HSD, honestly significant difference; POMT, protein O-mannosyltransferase; rt, rotated abdomen; tw, twisted; WT, wildtype control.

Dg is so far the only known target of protein O-mannosylation in *Drosophila* (37). POMT1 and POMT2 in *Drosophila* (also known as Rt and Tw) work together as an O-mannosyltransferase enzymatic complex, and mutations in either *rt* or *tw* cause the phenotype of “abdomen rotation”, a defect caused by misalignment of abdominal segments. Although Dg was shown to be a substrate of POMTs, Dg mutants do not have the abdomen rotation phenotype. However, defective O-mannosylation of Dg may result in a gain-of-function phenotype that is different from the phenotype of *Dg* loss-of-function alleles and thus whether the abdomen rotation phenotype of *POMTs* is caused by some gain-of-function effect of abnormally glycosylated Dg remains unknown. To shed light on this possibility, we analyzed the abdomen rotation phenotype of mutants carrying a combination of *POMT* and *Dg* alleles. *Drosophila Dg* null mutants are viable as adults (46), and they have no misalignment of abdominal segments (Fig. 1, *A* and *B*). Importantly, we found that *Dg* is not epistatic to *rt* and *tw* in producing the abdomen rotation phenotype, as it would be expected if Dg was the main functional substrate of POMTs in the pathway that controls abdomen development. *Dg-tw* and *Dg-rt* double mutants do not express functional Dg and hence would be expected to be similar to *Dg* single mutants if the main function of POMTs concentrates on Dg regulation. However, the double mutants show a prominent abdomen rotation, which indicates that this is not a *Dg* gain-of-function phenotype and that substrates other than Dg play important roles in the POMT pathway (Fig. 1, *A* and *B*). *Dg* and *POMTs* genetically interact, as the phenotype is stronger in *Dg-rt* and *Dg-tw* double mutants when compared with *rt* or *tw* single mutants, which suggests that Dg does contribute to the pathway, but not as a main player.

### *PTP69D* genetically interacts with *POMT2* in producing abdomen rotation

**Figure 2:**
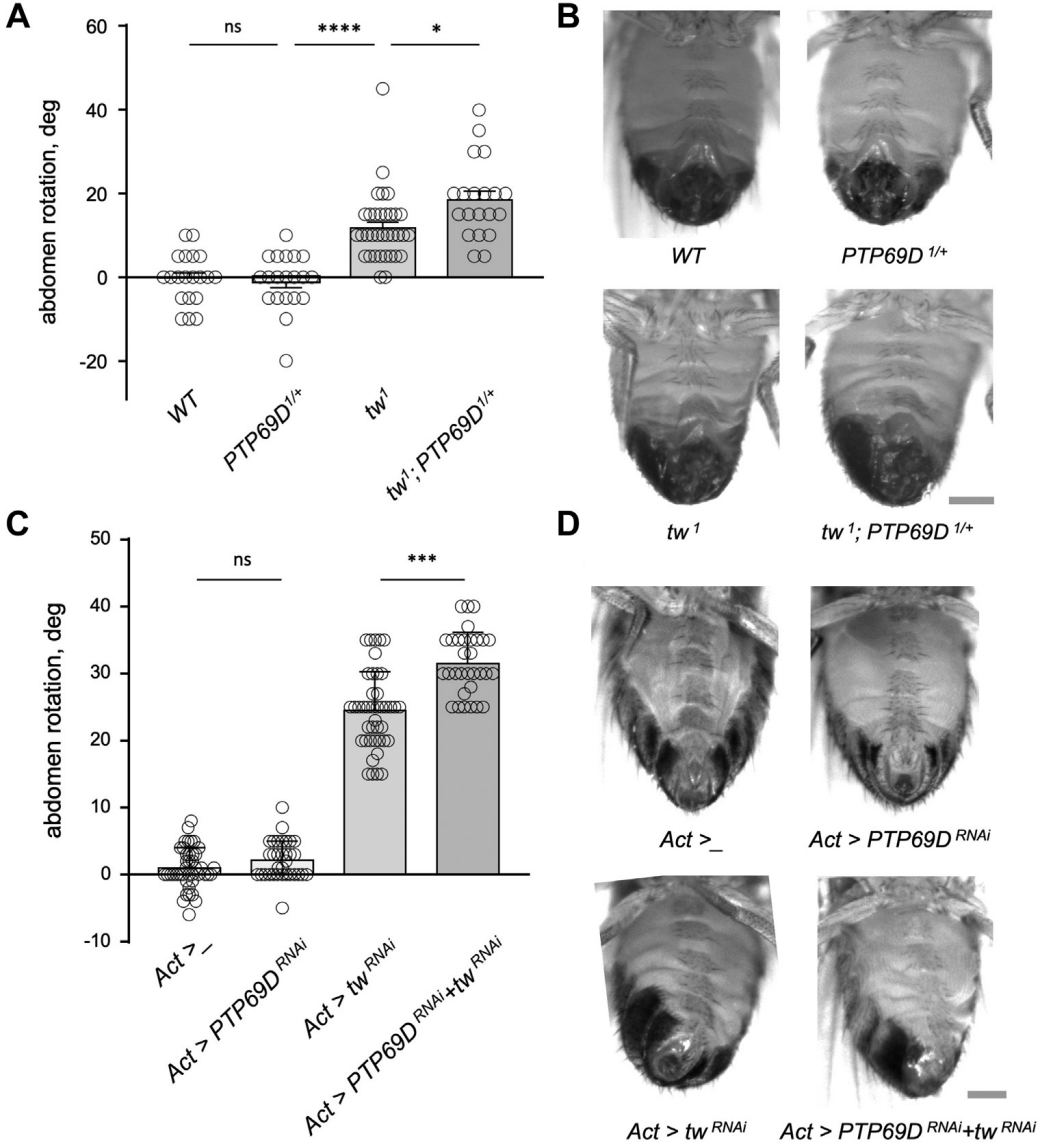
**Genetic interactions between *PTP69D* and *tw* in producing the abdomen rotation phenotype.***A*, one copy of *PTP69D*^*1*^ mutant allele enhances the abdomen rotation phenotype of *tw*^*1*^ mutants, whereas *PTP69D*^*1/+*^ heterozygotes do not have the phenotype by themselves. *B*, representative examples of the abdomen rotation phenotype of adult *Drosophila* males analyzed in *A*. *C*, RNAi-mediated knockdown genotypes of *PTP69D* and *tw* have synergistic interactions in producing abdomen rotation. KD was induced by expressing *UAS-PTP69D* and *UAS-tw-RNAi* constructs using *Act5C-Gal4* driver. Genotypes: *Act>_, Act5C-Gal4/+,* driver-only genotype (control). KD genotypes: *Act>PTP69D*^*RNAi*^*, Act5C-Gal4/UAS-PTP69D-RNAi. Act>tw*^*RNAi*^*, Act5C-Gal4/UAS-tw-RNAi. Act>PTP69D*^*RNAi*^*+ tw*^*RNAi*^*, Act5C-Gal4/UAS-PTP69D-RNAi UAS-tw-RNAi. D*, representative examples of the abdomen rotation phenotype of adult *Drosophila* females analyzed in *C*. *A* and *C*, positive and negative values denote clockwise counterclockwise rotation, respectively. Error bars are SEMs. Statistical significance was analyzed by one-way ANOVA with post hoc Tukey HSD test. The level of statistical significance: ns, not significant, *p* > 0.05; ∗, *p* < 0.05; ∗∗∗, *p* < 0.001; ∗∗∗∗, *p* < 0.0001. At least 20 flies were analyzed for each genotype (males in *A* and females in *C*). The scale bar represents 300 μm in *B* and *D*. HSD, honestly significant difference; *PTP69D*, protein tyrosine phosphatase 69D; tw, twisted.

With the aim to identify additional functional players of the *POMT*-mediated pathway, we tested the genetic interactions between *tw* and *PTP69D* (Fig. 2*A*). Our rationale was based on the fact that PTP69D is known to be involved in motoneuron axon guidance, and its mammalian homolog, RPTPζ, was found to be modified with O-mannosyl glycans (28, 33, 36). The addition of one copy of *PTP69D*^*1*^ null allele to *tw*^*1*^ hypomorphic mutants significantly enhanced the abdomen rotation phenotype, whereas *PTP69D*^*1*^ heterozygotes by themselves did not show any phenotype, which revealed a synergistic interaction between *PTP69D* and *tw*. To further confirm the genetic interactions between *tw* and *PTP69D*, we analyzed a combination of knockdown (KD) genotypes for *tw* and *PTP69D* generated by the expression of corresponding *UAS-RNAi* constructs. The *UAS-tw-RNAi* construct was previously shown to have a specific effect on *tw* and be able to induce a robust abdomen rotation (15, 16). The specific effect of *UAS-PTP69D-RNAi* expression was also previously confirmed in KD experiments (47). We found that the expression of *PTP69D-RNAi* construct by itself using a ubiquitous *Act5C-Gal4* driver did not cause any abdominal defects; however, its coexpression with *tw-RNAi* significantly enhanced the phenotype as compared with *tw* KD alone (Fig. 2*B*). Together, these results revealed that *POMTs* collaborate with *PTP69D* in the pathway affecting the alignment of abdominal segment.

### *PTP69D* collaborates with *POMT2* in wiring of sensory neuron connections

**Figure 3:**
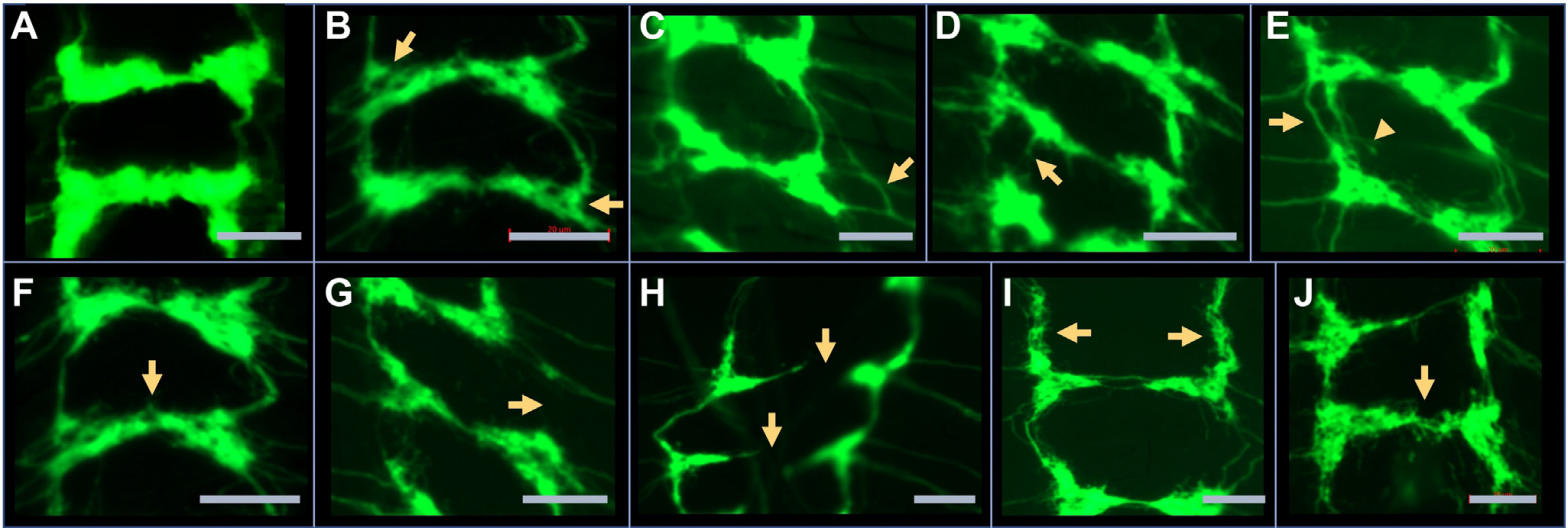
**Examples of different wiring defects of class IV sensory axons in the ventral ganglion of third instar *Drosophila* larvae.***A*, “WT” control (driver and marker only). *B*, split nodes. *C*, abnormal incoming axon bundles. *D*, ectopically branching commissural connection. *E*, split (*arrow*) and ectopically branching (*arrowhead*) longitudinal connections. *F*, split commissural connection. *G*, missing longitudinal connection. *H*, missing commissural connections. *I*, diffuse longitudinal connections. *J*, diffuse commissural connection. All panels: anterior is up; the scale bar represents 20 μm; axon connections are shown from the region of the third instar ventral ganglion corresponding to A2–A8 abdominal segments. Genotypes: *ppk-tdGFP/ppk-Gal4; ppk-Gal4/+* (*A*). *ppk-Gal4/ppk-tdGFP; ppk-Gal4/UAS-tw-RNAi, UAS-PTP69D-RNAi* (*B*, *E*, *F*, *I*, and *J*). *ppk-Gal4/ppk-tdGFP; ppk-Gal4/UAS-tw-RNAi* (*C*). *ppk-Gal4/ppk-tdGFP; ppk-Gal4/UAS-PTP69D-RNAi* (*D*, *G*, and *H*).

**Figure 4:**
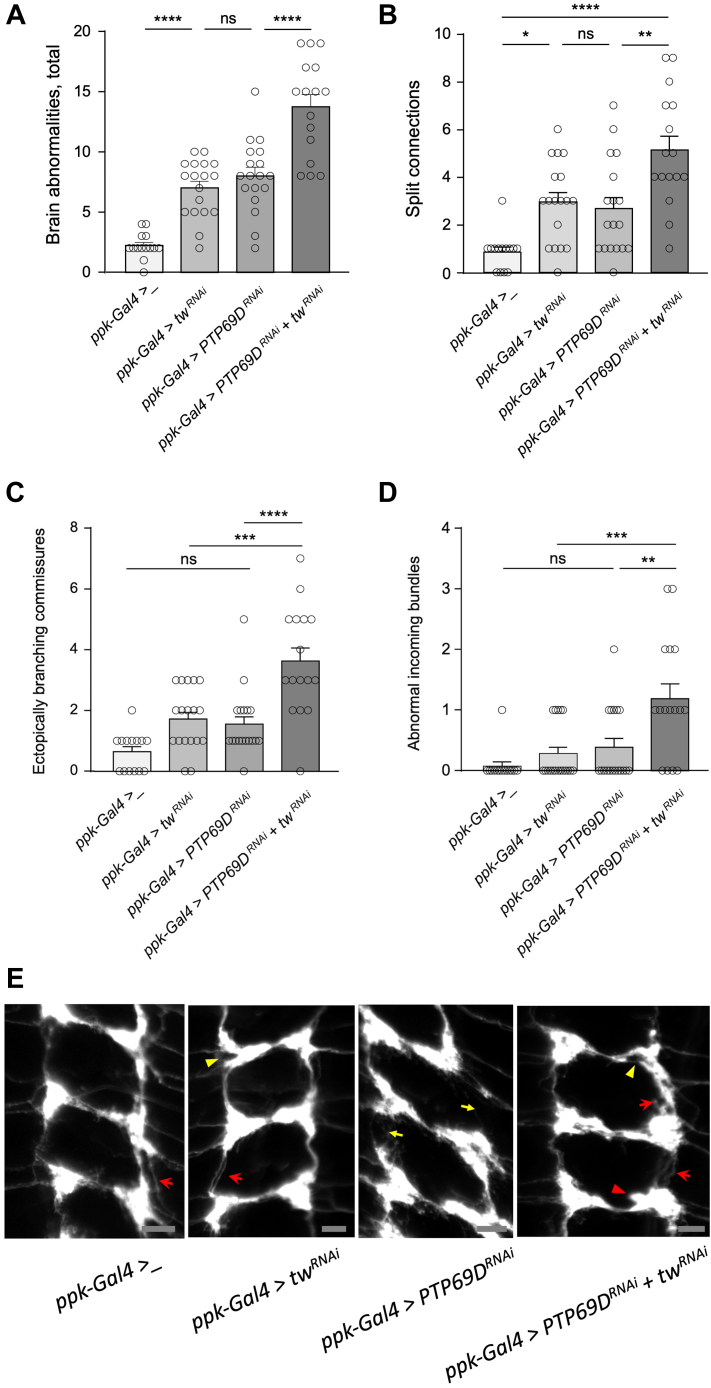
**Knocking down *PTP69D* or/and *tw* in class IV sensory neurons affects their axon wiring in the larval ventral ganglion.***A*, knockdown genotypes of *PTP69D* and *tw* have very similar wiring defects, while *PTP69D* - *tw* double KD results in further significant enhancement of the wiring phenotype. Different wiring defects were quantified per brain based on their effect on the ladder pattern of sensory axon connections. *B*–*D*, most prominent synergistic effect of *PTP69D* and *tw* KDs was found in the increase of split connections, ectopically branching commissures, and abnormal incoming axon bundles. *E*, representative images of class IV sensory axon connection in the larval ventral ganglion. *Red arrows*, split connections; *red arrowheads*, ectopically branching connections; *yellow arrows*, missing connections; *yellow arrowheads*, split nodes or node “bubbles.” Posterior region corresponding to abdominal segments is shown. Anterior is *top*. The scale bar represents 10 μm. All panels: at least 14 brains were analyzed for each genotype. Error bars are SEMs. Statistical significance was analyzed by one-way ANOVA with post hoc Tukey HSD test. The level of statistical significance: ns, not significant, *p* > 0.05; ∗, *p* < 0.05; ∗∗, *p* < 0.01; ∗∗∗, *p* < 0.001; ∗∗∗∗, *p* < 0.0001. Genotypes: *ppk-Gal4>_* ("wild-type" control), *ppk-Gal4/ppk-tdGFP; ppk-Gal4/+. ppk-Gal4>tw*^*RNAi*^ (*tw* KD genotype), *ppk-Gal4/ppk-tdGFP; ppk-Gal4/UAS-tw-RNAi. ppk-Gal4>PTP69D*^*RNAi*^ (*PTP69D* KD genotype) *ppk-Gal4/ppk-tdGFP; ppk-Gal4/UAS-PTP69D-RNAi. ppk-Gal4> PTP69D*^*RNAi*^*+ tw*^*RNAi*^ (*PTP69D* and *tw* double-KD)*, ppk-Gal4/ppk-tdGFP; ppk-Gal4/UAS-tw-RNAi UAS-PTP69D-RNAi.* HSD, honestly significant difference; *PTP69D*, protein tyrosine phosphatase 69D; tw, twisted.

Our previous experiments showed that *POMTs* are required for proper wiring of sensory axon termini in the larval ventral ganglion (40). Considering the genetic interactions between *POMTs* and *PTP69D* in producing abdomen rotation phenotype in adult flies, we hypothesized that these genes may also collaborate in establishing sensory neuron connections in the larval brain. We visualized class IV axon termini in the third instar larval central nervous system using *ppk-tdGFP* reporter (48) and analyzed their wiring pattern in genotypes with *tw* and *PTP69D* RNAi-mediated KD induced specifically in class IV dendritic arborization neurons by *ppk-Gal4* driver (40, 48). Our analysis of the “sensory ladder” revealed several types of wiring defects, including (i) ectopically branching connections, (ii) missing connections, (iii) diffused connections; (iv) split connections; (v) split nodes or node “bubbles”; and (vi) abnormal incoming bundles (Fig. 3). The effects of *tw* and *PTP69D* KD on sensory axons was quantified by counting these wiring defects per brain (Fig. 4). We found that individual KD of *tw* or *PTP69D* resulted more frequently in similar types of wiring defects, such as split and ectopically branching connections, and abnormal incoming bundles (Fig. 4, *A* and *B*). Double KD of *tw* and *PTP69D* significantly increased the total number of defects (Fig. 4*A*). As compared with individual KDs, the *tw-PTP69D* double KD genotype showed significantly increased number of split connections and ectopically branching commissural connections, whereas the phenotype of abnormal incoming bundles was especially enhanced, showing synergistic interactions between *tw* and *PTP69D* (Fig. 4, *B*–*D*). Taken together, our results uncovered that *PTP69D* is required for wiring of class IV sensory axons, revealed that *PTP69D* genetically interacts with *tw* in this pathway, and showed synergistic interactions in producing wiring defects of sensory axons.

### Ectopic expression of *POMTs* and dominant-negative *PTP69D* show synergistic interactions in sensory neurons

**Figure 5:**
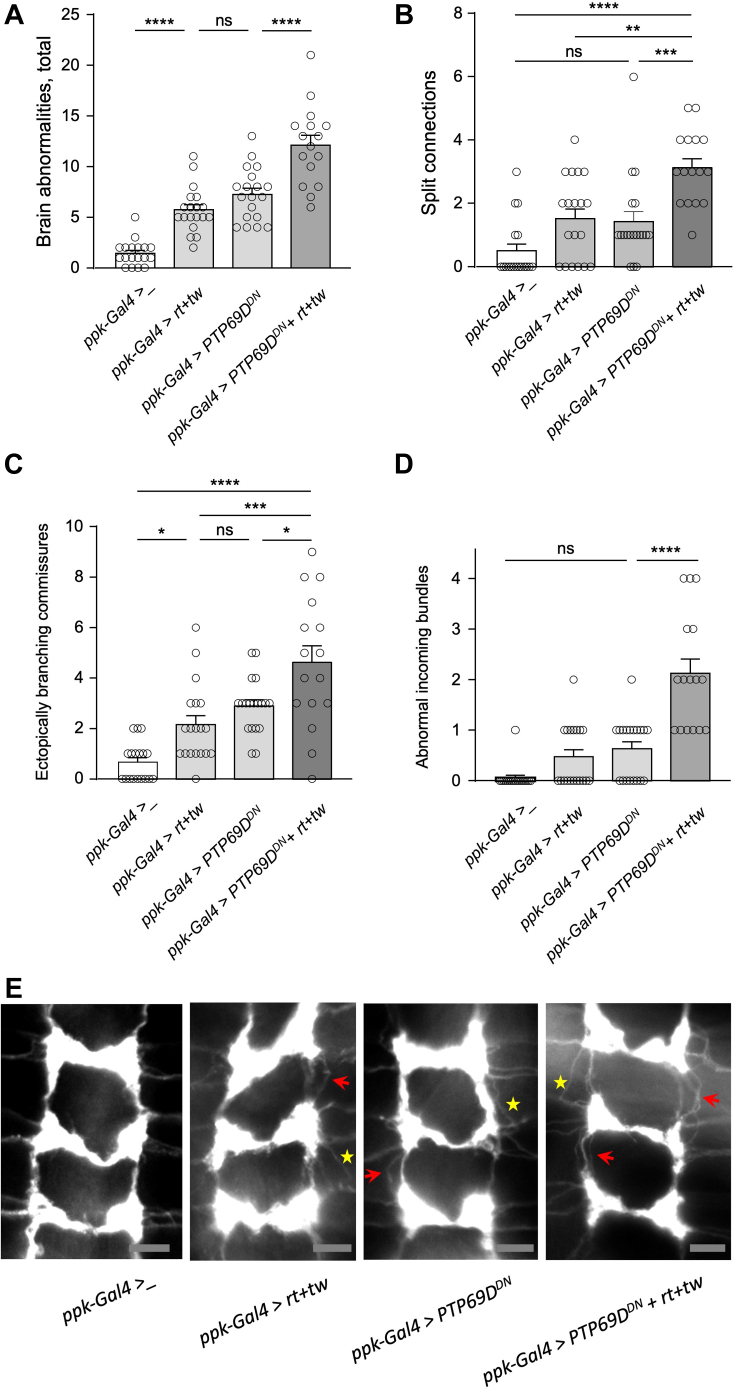
**Ectopic expression of dominant-negative PTP69D induces wiring defects of sensory axons and shows synergistic interactions with coexpression of POMTs.***A*, ectopic expression of PTP69D^DN^, a dominant-negative form of PTP69D, and coexpression of *rt* and *tw* in sensory neurons induce axon wiring defects. Combining the expression of *PTP69D*^*DN*^ with *rt* and *tw* causes further significant increase in wiring defects. *B*–*D*, synergistic effect of co-overexpression of *PTP69D*^*DN*^ with *rt* and *tw* on the phenotypes of split connections, ectopically branching commissures, and abnormal incoming axon bundles. *E*, representative images of class IV sensory axon connection in the larval ventral ganglion. *Red arrows*, split connections; *stars*, abnormal incoming bundles. Posterior region corresponding to abdominal segments is shown. Anterior is *top*. The scale bar represents 10 μm. All panels: at least 16 brains were analyzed for each genotype. Error bars represent SEMs. Statistical significance was analyzed by one-way ANOVA with post hoc Tukey HSD test. The level of statistical significance: ns, not significant, *p* > 0.05; ∗, *p* < 0.05; ∗∗, *p* < 0.01; ∗∗∗, *p* < 0.001; ∗∗∗∗, *p* < 0.0001. Genotypes: *ppk-Gal4>_* (control), *ppk-Gal4/+; ppk-tdGFP/+. ppk-Gal4>rt + tw* (*rt* and *tw* co-overexpression genotype), *ppk-Gal4/UAS-rt UAS-tw; ppk-tdGFP/+. ppk-Gal4>PTP69D*^*DN*^*(PTP69D* dominant-negative genotype*) PTP69D*^*DN*^*/+; ppk-Gal4/+; ppk-tdGFP/+. ppk-Gal4> PTP69D*^*DN*^*+ rt+tw* (*PTP69D dominant-negative* and *rt and tw co-overexpression* genotype), *PTP69D*^*DN*^*/+; ppk-Gal4/UAS-rt UAS-tw; ppk-tdGFP/+.* HSD, honestly significant difference; *PTP69D*, protein tyrosine phosphatase 69D; rt, rotated abdomen; tw, twisted.

To further validate the involvement of PTP69D in establishing sensory neuron connectivity, we analyzed the effect of a dominant-negative form of PTP69D, PTP69D^DN^, which lacks the intracellular domain (also known as PTP69DΔintra) (49), on wiring of sensory axons (Fig. 5). The expression of PTP69D^DN^ in class IV sensory neurons largely phenocopied the effect of *PTP69D* KD (Fig. 5, *A*–*D*). Interestingly, we found that co-overexpression of PTP69D^DN^ with POMTs, Rt and Tw, significantly enhanced the phenotype of PTP69D^DN^ (Fig. 5*A*), showing prominent enhancement of the phenotypes of abnormal incoming axon bundles and split connections (Fig. 5, *C* and *D*). The dominant-negative form of PTP69D is thought to exert its effect by sequestering RPTP ligand(s) (49). The synergistic effect of overexpression of POMTs on the phenotype of PTP69D^DN^ suggests that POMTs potentiate binding between PTP69D^DN^ and the ligand(s). Overexpression of POMTs alone also resulted in some increase of wiring defects, suggesting that it may be associated with ectopic O-mannosylation that interferes with normal regulation of axon connectivity. Further experiments are required to test this possibility.

### *POMT2* and *PTP69D* mutant alleles can show antagonistic interactions in producing axon wiring phenotypes

**Figure 6:**
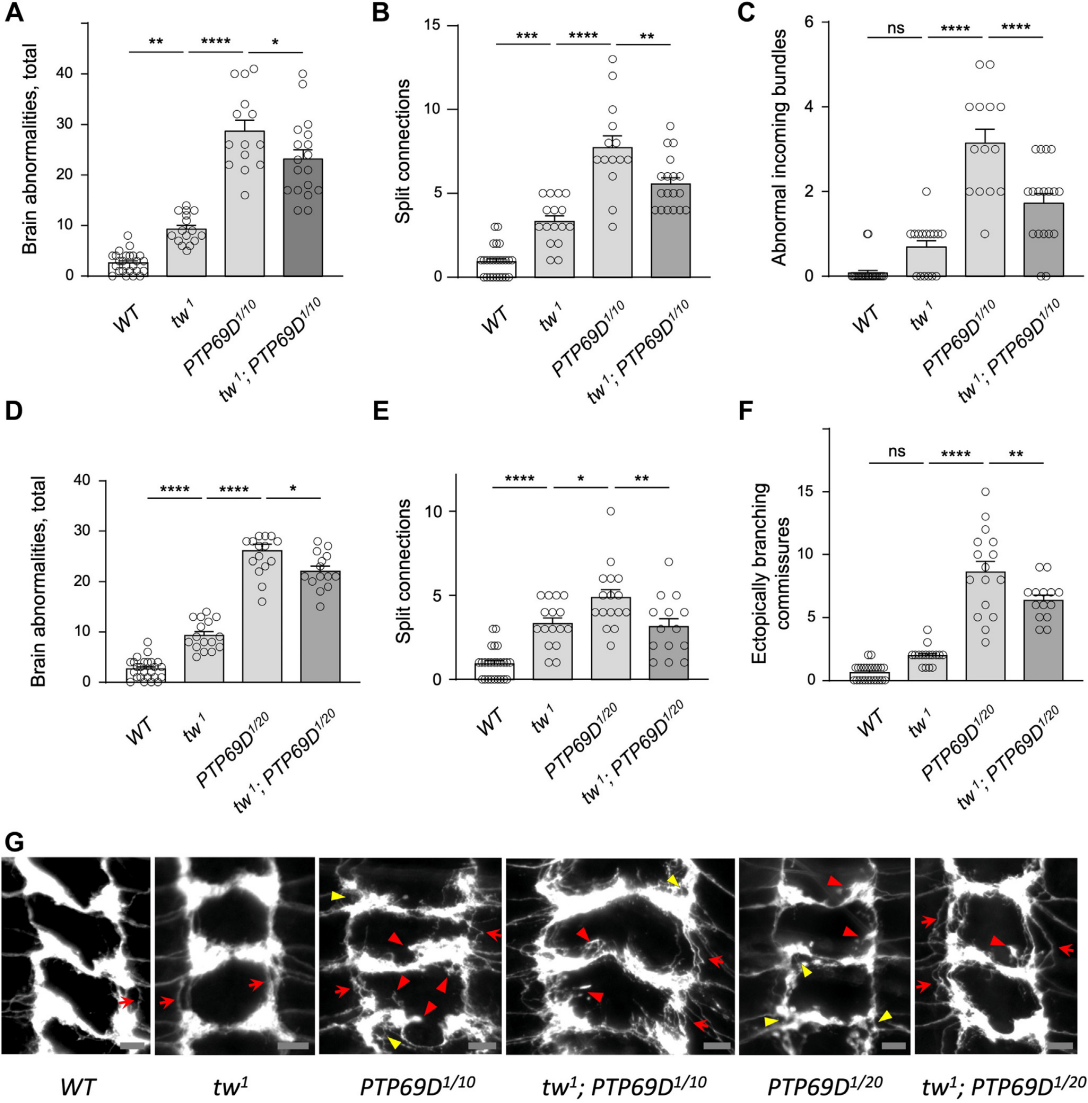
**Downregulation of *PTP69D* by mutant alleles induces wiring defects of sensory axons and shows antagonistic interaction with *tw* mutation.***A*–*C*, *PTP69D*^*1/10*^ mutants have conspicuous defects of sensory axon wiring, whereas these defects are ameliorated in *tw*^*1*^; *PTP69D*^*1/10*^ double mutants. *A*, analysis of total number of wiring defects per brain in WT larvae (*WT*), *tw*^*1*^ mutants, *PTP69D*^*1/10*^ mutants, and *tw*^*1*^*PTP69D*^*1/10*^ double mutants. *B* and *C*, *tw*^*1*^ and *PTP69D*^*1/10*^ show prominent antagonistic interaction in producing phenotypes of split connections (*B*) and abnormal incoming axon bundles. *D*–*F*, comparing wiring defects in *tw*^*1*^, *PTP69D*^*1/20*^, and their combination: total number of wiring defects per brain (*D*); the phenotype of split connections (*E*); and ectopically branching commissural connections (*F*). The wiring defects are significantly ameliorated in *tw*^*1*^; *PTP69D*^*1/20*^ double mutants as compared with *PTP69D*^*1/20*^ mutants, which indicates antagonistic genetic interactions between *PTP69D* and *tw*. *G*, representative images of class IV sensory axon connection in the larval ventral ganglion. *Red arrows*, split connections; *red arrowheads*, ectopically branching connections; *yellow arrowheads*, split nodes or node “bubbles". Posterior region corresponding to abdominal segments is shown. The scale bar represents 10 μm. All panels: error bars represent SEMs; statistical significance was analyzed by one-way ANOVA with post hoc Tukey HSD test. The level of statistical significance: ns, not significant, *p* > 0.05; ∗, *p* < 0.05; ∗∗, *p* < 0.01; ∗∗∗, *p* < 0.001; ∗∗∗∗, *p* < 0.0001. Genotypes: *WT* (control), *ppk-tdGFP/+. tw*^*1*^ (*tw* mutant genotype), *tw*^*1*^*; ppk-tdGFP/+. PTP69D*^*1/10*^ (*PTP69D* mutant genotype), *ppk-tdGFP/+; PTP69D*^*1*^*/PTP69D*^*10*^*. tw*^*1*^*; PTP69D*^*1/10*^ (*PTP69D* and *tw* double mutant genotype), *tw*^*1*^*; ppk-tdGFP/+; PTP69D*^*1*^*/PTP69D*^*10*^*. PTP69D*^*1/20*^ (*PTP69D* mutant genotype)*, ppk-tdGFP/+; PTP69D*^*1*^*/PTP69D*^*20*^*. tw*^*1*^*; PTP69D*^*1/20*^ (*PTP69D* and *tw* double mutant genotype)*, tw*^*1*^*; ppk-tdGFP/+; PTP69D*^*1*^*/PTP69D*^*20*^*.PTP69D*, protein tyrosine phosphatase 69D; tw, twisted.

RPTPs can function both cell-autonomously and cell-nonautonomously (50, 51), which suggests that the effect of *PTP69D* global downregulation may be different from its downregulation limited to sensory neurons. Similarly, POMTs may potentially affect wiring of sensory neurons both cell-autonomously and nonautonomously by modifying proteins in sensory neurons, as well as in other cells that regulate axon wiring *via* cell signaling or cell adhesion. Thus, we decided to test if ubiquitous downregulation of these genes would have a different effect on axon connectivity, as compared with their downregulation restricted to sensory neurons. To this end, we created genotypes that included mutant alleles of these genes (52). *PTP69D*^*1*^ null allele has a deletion within the gene region and is embryonic lethal as homozygous (42). Thus, we created hypomorphic genotypes combining mutant alleles of different strength, including *PTP69D*^*10*^ (a hypomorphic allele with a missense mutation in the second immunoglobulin-like domain) and *PTP69D*^*20*^ (a hypomorphic allele with a missense mutation in the first protein tyrosine phosphatase domain) (52). We found that *tw*^*1*^ mutants have increased number of wiring defects, as compared with WT controls, whereas *PTP69D*^*1*/*10*^ and *PTP69D*^*1*/*20*^ mutants showed significantly more severe wiring phenotype than *tw*^*1*^ mutants (Fig. 6). Unexpectedly, our results also revealed that *tw*^*1*^ ameliorated the phenotype of *PTP69D* mutants, which unveiled an antagonistic mode of *twPTP69D* genetic interactions. These results suggest that POMTs may have other molecular targets that affect wiring of sensory axons and can promote or antagonize PTP69D functions.

### *POMT* mutants show stronger wiring phenotype than *Dg* mutants

**Figure 7:**
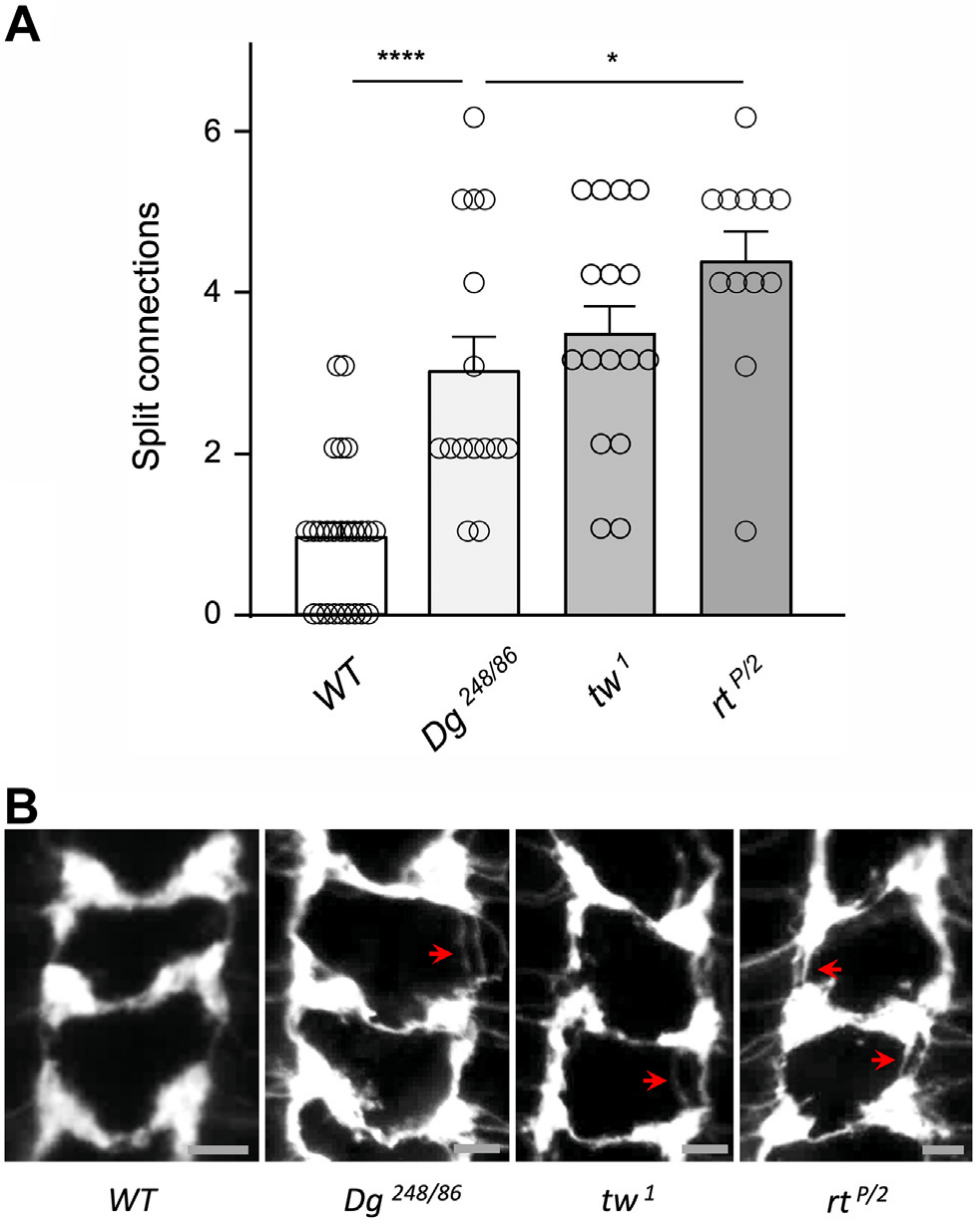
**Sensory axon wiring phenotypes of *Dg* and *POMT* mutants.***A*, *POMT1* hypomorphic mutants (*rt*^*P/2*^) have stronger phenotype of sensory axon connectivity than *Dg* null mutants (*Dg*^*248/86*^). The *Dg* phenotype is comparable to the phenotype of *tw*^*1*^, a weak hypomorphic *POMT2* mutant. *B*, representative images of sensory axon connection in the larval ventral ganglion. *Red arrows*, split connections. Region corresponding to abdominal segments is shown. Anterior is *top*. The scale bar represents 10 μm. *A*, error bars are SEMs; statistical significance was analyzed by one-way ANOVA with post hoc Tukey HSD test. Genotypes: *WT* (control), *ppk-tdGFP/+. tw*^*1*^ (*tw* mutant), *tw*^*1*^*; ppk-tdGFP/+. Dg*^*248/86*^ (*Dg* mutan*t*), *ppk-tdGFP Dg*^*248*^*/Dg*^*86*^*. rt*^*P/2*^ (*rt* mutant)*, ppk-tdGFP/+; rt*^*P*^*/rt*^*2*^*.* Dg, dystroglycan; HSD, honestly significant difference; POMT, protein O-mannosyltransferase; rt, rotated abdomen; tw, twisted.

To shed light on the relationship between *Dg* in *POMTs* in the regulation of axon connectivity, we compared the sensory axon wiring phenotypes of these genes. The effect of *Dg* on axon wiring has not been previously analyzed, so we analyzed *Dg* loss-of-function mutants using a heteroallelic combination of null alleles (*Dg*^*248/86*^). *Dg* null mutants showed a relatively mild wiring phenotype of sensory axons, which was similar to that of *tw*^*1*^, a weak hypomorphic allele of *POMT2* (Fig. 7). We also analyzed the wiring phenotype of *POMT1* mutants with a heteroallelic combination of strong hypomorphic alleles (*rt*^*P/2*^), which revealed that *POMT1* hypomorphs have a stronger phenotype than *Dg* null mutants (Fig. 7). This analysis indicated that *Dg*, unlike *POMTs* and *PTP69D*, does not play a prominent role in axon connectivity, although it still contributes to proper wiring of axon termini. This suggested that the function of *POMTs* in establishing sensory axon connectivity largely depends on POMT targets other than Dg, which is in agreement with the results of genetic interactions between *POMTs* and *Dg* in producing abdomen rotation phenotype (Fig. 1). Taken together, these results further supported the scenario that *POMTs* and *PTP69D* collaborate in the pathway that is largely independent of *Dg*.

### PTP69D extracellular domain is a target of POMT-dependent O-mannosylation

**Figure 8:**
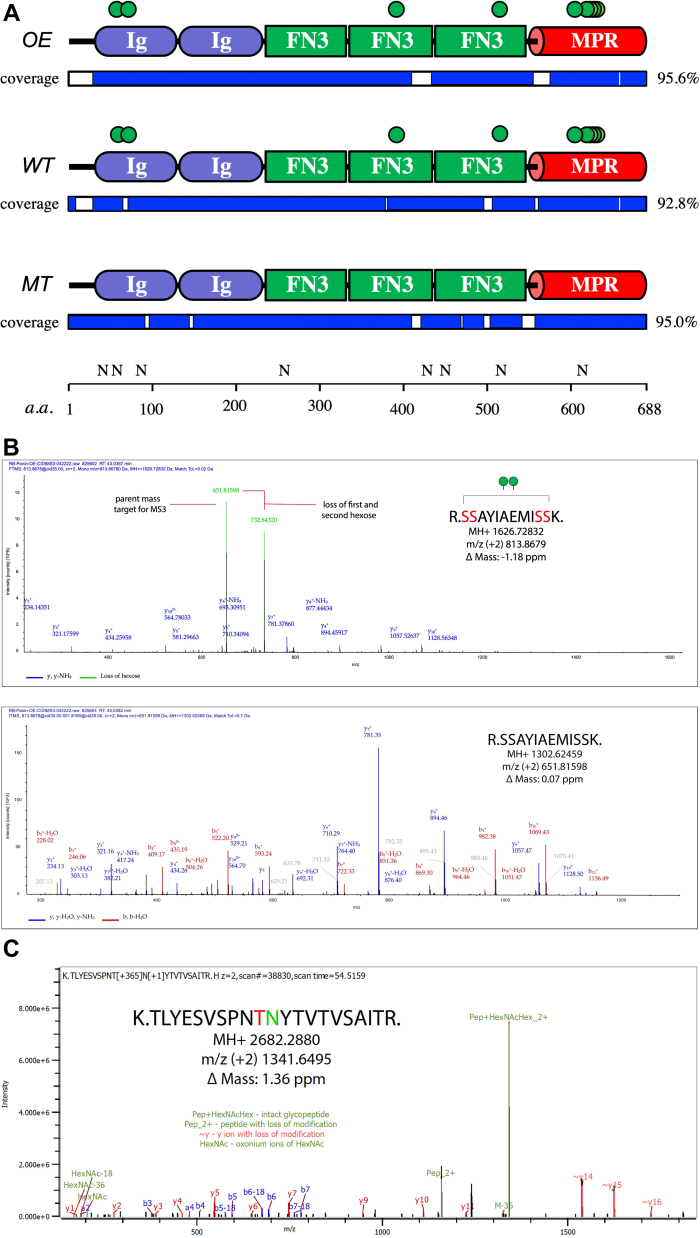
**Glycosylation of PTP69D extracellular region revealed by mass spectrometry.***A*, summary of glycosylation sites identified on PTP69D-EC-FLAG construct expressed *in vivo* in *rt*^*2/P*^ mutants (MT), *WT*, or in the genotype with co-overexpression of Rt and Tw (OE). Amino acid numbers are shown according to the PTP69D-PA isoform sequence (FlyBase ID: FBgn0014007). Structural features depicted: FN3, fibronectin type 3 domain; Ig, immunoglobulin-type domain, MPR, membrane proximal region. Coverage indicates the regions that were represented by peptides identified by MS. , O-hexose modifications identified with high confidence. N, locations on N-linked glycans (invariably found in all three samples). *B*, example of CID neutral loss trigger on O-mannosylated glycopeptide. MS^2^ spectrum (*top*) shows intense signal for parent ion sans sugar modifications. Isolation and MS^3^ fragmentation of that peak reveals excellent b and y ion series to confirm peptide sequence (*bottom*). *C*, example of sHCD spectrum following PNGase F/A treatment and POMGnT1 extension illustrating the site of O-mannose modification as well as the site of N-linked glycosylation (PNGase F/PNGase A reaction cleaves the entire N-linked glycan leaving behind a deamidated asparagine residue, which results in N to D conversion). CID, collision-induced dissociation; OE, overexpression; OMGnT1, protein O-mannose β1,2-N-acetylglucosaminyltransferase; PTP69D, protein tyrosine phosphatase 69D; rt, rotated abdomen; sHCD, stepped high-energy collision dissociation; tw, twisted.

The genetic interactions between *POMTs* and *PTP69D* indicate that they participate in the same functional pathway, suggesting that PTP69D may represent an important target of POMTs in the axon wiring pathway, and that POMTs may directly affect PTP69D activity by modifying it with O-linked mannose. To test this hypothesis, we generated a *UAS-PTP69D-EC-FLAG* construct encoding a FLAG-tagged extracellular domain of PTP69D (see Experimental procedures section). We expressed this construct *in vivo* by the *UAS-GAL4* system (53) using a ubiquitous driver (*Act5C-Gal4*). The construct was expressed in three different genetic backgrounds, *WT*, *POMT1* mutant (*rt*^*2/P*^), and in a genetic background with ectopic upregulation of POMTs by co-overexpression of *UAS-tw* and *UAS-rt* transgenes (designated as *WT*, *MT*, and *OE*, respectively). The PTP69D-EC-FLAG protein was purified from *Drosophila* and analyzed by mass spectrometry for potential glycosylation. The analysis showed similar peptide coverage for all three samples (greater than 92% for each). All three proteins were found to bear eight N-linked glycans at the same modification sites (Fig. 8, Table 1 and Fig. S1). O-linked hexose was found on PTP69D-EC-FLAG purified from OE and WT backgrounds but not on the protein expressed in *rt*^*2/P*^ mutants. Nine O-linked hexoses were identified on PTP69D from OE and WT backgrounds (Fig. 8, *A* and *B*, Table 1 and Fig. S2). The identity of hexose modifications was confirmed by both collision-induced dissociation (CID) pseudoneutral loss of a hexose triggered MS^3^ and stepped high-energy collision dissociation (sHCD) (Fig. 8*B*). In order to map hexoses to specific sites and demonstrate that the O-hexose was in fact an O-mannose, we treated tryptic peptides with POMGnT1 (protein O-mannose β1,2-*N*-acetylglucosaminyltransferase) and UDP-GlcNAc and reanalyzed by sHCD. POMGnT1 is a mammalian glycosyltransferase that specifically modifies O-linked mannose with β1,2-linked GlcNAc (54) and thus, its activity toward glycopeptides indicates the presence of not extended O-mannosyl glycans. This approach allowed us to confirm that at least two O-hexoses found on the two most abundant glycopeptides were indeed modifications with O-mannose (Figs. 8*C*, and S3). Furthermore, this allowed us to unambiguously assign the O-mannose modification on T515, which was difficult to do by other approaches (Fig. 8*C*). The absence of O-linked hexoses on PTP69D isolated from *POMT1* mutants (MT sample) indicated that these modifications depend on POMT activity, which suggested that all identified hexose modifications represent O-linked mannose added by POMTs.

**Table 1:** Identified N-linked glycans and O-hexose modifications on PTP69D-EX-FLAG construct expressed in *rt*^*2/P*^ mutant (MT), WT, and *rt + tw* OE genetic backgrounds

Glycopeptide	Modification type	MT	WT	OE
**N**(40)VSLECASENEAVAWK	N-glycan	+	+	+
LG**N**(58)QTINK	N-glycan	+	+	+
LGNQ**T**(60)INK^a^	O-hexose	−	+	+
**T**(72)EPLK^a^	O-hexose	−	+	+
SNDDGSEN**N**(85)DSQDFIK	N-glycan	+	+	+
IYL**N**(255)WTVNDGNDPIQK	N-glycan	+	+	+
VIEEAIYQQN**S**(391)R	O-hexose	−	+	+
TCGPWSENV**N**(429)GTTMDGVATK	N-glycan	+	+	+
PTNLSIQCHHD**N**(451)VTR	N-glycan	+	+	+
TLYESVSPN**T**(515)NYTVTVSAITR^b^	O-hexose	−	+	+
TLYESVSPNT**N**(516)YTVTVSAITR	N-glycan	+	+	+
LNIAT(605)YQEVHS(611)DNVT(615)R^c^	O-hexose	−	+	+
LNIATYQEVHSD**N**(613)VTR	N-glycan	+	+	+
**SS**(617/618)AYIAEMI**SS**(626/627)^b^	O-hexose	−	+	+

Eight of the nine O-hexose sites were mapped to a specific residue; + and – indicate that the modification was identified, or no modification was found by MS analyses, respectively.

a Identified by sHCD only (all other modifications were identified by both fragmentation methods).

b Hexose was found to be extended with GlcNAc after *in vitro* treatment by POMGnT1, which confirmed the identity of the hexose as mannose. The sites of modifications that were determined unambiguously are shown in bold, numbers indicate positions of glycosylated amino acids within PTP69D protein sequence. The mass spectrometry proteomics data have been deposited to the ProteomeXchange Consortium *via* the PRIDE partner repository, the dataset identifier PXD034563.

c Hexose was identified, but the attachment site was not unambiguously determined (potential sites are underlined).

## Discussion

*POMT* mutations in mammals cause abnormal glycosylation of Dg and result in severe muscular dystrophies, such as Walker–Warburg syndrome and muscle–eye–brain disease that are associated with prominent neurological defects, including cobblestone lissencephaly and other brain malformations (7, 19, 20, 22, 24, 25). These neurological phenotypes are difficult to explain solely by defects in Dg functions; however, other functional substrates of POMTs in the nervous system remain unknown (7). Here, we employed *Drosophila* as a model for evolutionarily conserved functions of POMTs (6, 15, 16, 37, 40) to shed light on the involvement of Dg and possibly other POMT substrates in the phenotypes caused by POMT mutations. We analyzed *tw* and *Dg* single and double mutants and compared their abdomen rotation phenotypes. The genetic interactions revealed in these experiments suggested that these phenotypes are mainly caused by POMT substrates other than Dg. We identified *PTP69D* as a gene required for the wiring of sensory axons and found that it strongly interacts with *tw* in producing abdomen rotation and sensory axon defects. Although the scenarios placing *POMTs* and *PTP69D* in separate functional pathways impinging on sensory axon wiring are also conceivable, taken together, our data strongly suggest that these genes collaborate while working in the same pathway. Our results uncovered the first evidence of functional interactions between *POMTs* and *RPTPs*, suggesting that similar interactions may exist in mammals. *PTP69D* is known to play important roles in *Drosophila* neural development, regulating axon guidance of motoneurons, targeting photoreceptor axons, and establishing axon connections of the giant fiber neurons (42, 44, 45); however, its function in the wiring of sensory axons was not previously investigated, and it is revealed for the first time in our analysis of *PTP69D* mutants. In addition to the analyses of mutant genotypes, we also confirmed this function by the expression of a dominant-negative form of PTP69D with truncated intracellular phosphatase domains. Interestingly, coexpression of *PTP69D*^*DN*^ with *POMTs* significantly enhanced the axon wiring phenotype, suggesting that POMTs may potentiate the effect of PTP69D^DN^ by promoting its ligand interactions, possibly *via* direct modification of PTP69D^DN^ with O-mannose. Other conceivable scenarios may involve some unknown POMT substrates that interfere with PTP69D or contribute to axon wiring independently. Testing these possibilities will require further investigation.

Interestingly, we found that the phenotype of *PTP69D* mutations was ameliorated by *tw*^*1*^ in double mutants, which revealed that *PTP69D* and *POMTs* can have antagonistic interactions. This result can be potentially explained by the effect of POMTs on some other substrates involved in sensory axon wiring. Previous experiments revealed that several RPTPs can participate in the same functional pathway regulating axons in other developmental contexts, while showing synergistic, redundant, or antagonistic relationship (55). POMTs can potentially affect the function of other RPTPs, which may explain the complex genetic interactions between *POMTs* and *PTP69D* revealed in our experiments. Furthermore, RPTPs can have both cell-autonomous and cell-nonautonomous functions (50), which may also contribute to the complex genetic interactions unveiled in our experiments. Interestingly, although our results indicated that Dg does not play a major role in sensory axon wiring, it has some contribution to the pathway, which is supported by axon wiring defects in *Dg* mutants (Fig. 7). This conclusion is also consistent with the genetic interactions between *Dg* and *POMTs* in producing abdomen rotation phenotype (Fig. 1). Although the effect of *Dg* on axon connectivity is relatively mild, it may modulate *POMT–PTP69D* genetic interactions and contribute to their outcome. All these scenarios, however, remain speculative, and further studies are required to elucidate the molecular and cellular mechanism of *POMTRPTP* functional interactions.

The genetic interactions between *POMTs* and *PTP69D* suggested that PTP69D is a functional target of POMT-mediated O-mannosylation. RPTPζ, a mammalian homolog of PTP69D that is broadly expressed in the brain and involved in the formation of perineural nets, is known to carry O-mannosyl glycans (28, 29). Although it was hypothesized that POMTs may mediate the addition of O-mannose to RPTPζ (7), so far no direct evidence of POMT-dependent O-mannosylation of RPTPζ has been obtained. Our mass spectrometry–based analyses revealed that the extracellular part of PTP69D is modified with O-linked hexose in genotypes with normal or elevated level of the activity of POMT, but not in *POMT1* mutants (which also lack the ability to modify Dg (37)), whereas PTP69D was similarly modified with eight N-linked glycans in all analyzed genotypes (Fig. 8*A* and Table 1). The O-hexose modifications were confirmed to be O-mannose for two of the nine sites by *in vitro* extension with POMGnT1 (Figs. 8*C* and S3). These sites were found on the two most abundant glycopeptides, whereas the other sites were not amenable to this analysis because of the limited amount of material. Previous comprehensive analysis of the *Drosophila* O-glycoproteome identified three types of O-linked hexose modifications, including O-linked mannose, glucose, and fucose (56). However, all known glycosyltransferases that can modify *Drosophila* proteins with O-linked hexose other than mannose, such as the O-glucosyltransferase Rumi, and O-fucosyltransferases 1 and 2, show strict substrate preferences for protein motifs that are not present in PTP69D (57, 58, 59), which suggests that all O-linked hexose modifications of PTP69D represent O-mannose. This conclusion is consistent with our analysis that indicated that all O-hexose modifications of PTP69D construct depend on *rt* activity (Fig. 8*A*). It is interesting to note that the sites of O-mannosylation identified on PTP69D belong to protein domains that are not similar to protein regions of Dg known to be modified by POMTs (17, 37), which highlights the complexity of POMT substrate recognition and expands the number of potential POMT substrates in animal proteomes. Taken together, our results indicate that PTP69D is a novel substrate of POMT-mediated O-mannosylation. The fact that the upregulation of POMTs did not result in new modification sites (Fig. 8*A*) suggests that the POMTs modify PTP69D with high specificity, and that the identified O-mannose sites correspond to endogenous PTP69D modification sites. How these different modifications affect PTP69D function and which of them are functionally more important will be pivotal questions for future studies. Finally, considering evolutionary conservation of RPTPs, as well as the conservation of POMTs, our findings suggest that mammalian RPTPs, such as RPTPζ and possibly other RPTPs, are also substrates of POMTs, and that POMTs are important effectors of RPTP functions in mammalian organisms, which may underpin neurological defects of dystroglycanopathies caused by *POMT* mutations. This important implication warrants further investigation.

## Experimental procedures

### *Drosophila* strains

Genetic strains with mutant alleles, Gal4 drivers, and transgenic constructs used in the study were obtained from the Bloomington *Drosophila* Stock Center (*Dg*^*86*^*; Dg*^*248*^, *rt*^*P*^, *rt*^*2*^, *tw*^*1*^, *PTP69D*^*1*^, *PTP69D*^*10*^, *PTP69D*^*20*^, *ppk-Gal4*, *Act5C-Gal4*, *ppk-tdGFP*, *PTP69D-RNAi*), Vienna *Drosophila* Resource Center (*UAS-tw-RNAi*), and Kyoto Stock Center (*UAS-PTP69DΔintra*). The mutant alleles were previously characterized; briefly, *tw*^*1*^ is a hypomorphic allele of *POMT2*; *rt*^*2*^ and *rt^P^* are strong hypomorphic alleles of *POMT1* that are close to amorphs; *Dg*^*86*^ and Dg^248^ are null alleles; *PTP69D*^*1*^ is a null allele, and *PTP69D*^*10*^ and *PTP69D*^*20*^ are hypomorphic alleles (16, 52, 60, 61). *UAS-rt* and *UAS-tw* transgenes were previously described (16). *UAS-PTP69D-EC-FLAG* transgenic strain was generated by a standard P-element–mediated germline transformation. The genetic background of all strains was *Canton S w-.*

### Molecular cloning

Full-length *PTP69D complementary DNA* clone (RE06719) was obtained from *Drosophila* Genomics Resource Center. The *UAS-PTP69D-EC-FLAG* construct for *in vivo* transgenic expression was generated using standard molecular biology techniques essentially according to previously designed protocols (see Experimental procedures section of Supporting Information) (62). The construct encodes the PTP69D-EC-FLAG protein including the entire extracellular region of PTP69D truncated 20 amino acids upstream of a putative juxtamembrane proteolytic cleavage site (52) that was fused to a C-terminal 3FLAG tag.

### *In vivo* expression and purification of PTP69D-EC-FLAG

The expression and purification of PTP69D-EC-FLAG was carried out essentially according to the previously published protocol (37, 63). Briefly, transgenic *Drosophila* strains carrying genome-integrated *UAS-PTP69D-EC-FLAG* construct were obtained by P-element–mediated germline transformation. The *in vivo* expression of the *UAS-PTP69D-EC-FLAG* transgene was induced using the UAS-GAL4 system (64). The PTP69D-EC-FLAG protein was purified from pupae using anti-FLAG M2 agarose (Sigma). For each purification experiment, 80 to 100 *Drosophila* were collected and lysed in 1.5 to 2 ml of lysis buffer (50 mM Tris–HCl, pH 7.4, 150 mM NaCl, 1% Triton X-100) including cocktail of protease inhibitors (cOmplete; Roche). Following preclearing of the lysate by centrifugation, the supernatant was incubated with 30 to 40 μl of FLAG beads overnight at 4 °C with nutation. The agarose beads were then washed extensively with the lysis buffer, and the purified PTP69D-EC-FLAG protein bound to beads was directly used in further experiments.

### Abdomen rotation phenotype

The abdomen rotation phenotype was analyzed as previously described (16). Briefly, adult *Drosophila* flies were collected on the day of eclosion and aged for 2 to 5 days before scoring the phenotype. This ensured that the cuticle of the abdomen is fully hardened and decreased possible effect of muscle contractions on the abdomen shape during the scoring of abdomen rotation. The angle of the rotation was measured using a Zeiss Stemi stereomicroscope with a protractor glass insert installed in the eyepiece. Flies were immobilized, and the abdomen rotation phenotype was analyzed using the posterior view of the abdomen. Angle of misalignment of the last abdominal segment relative to the anterior end of the abdomen was recorded as the measure of the phenotype.

### Larval brain immunostaining and analyses of sensory axon wiring phenotypes

The dissection and immunostaining of larval brains was carried out as previously described (40). Briefly, class IV axon connections in the ventral ganglion were labeled by the expression of ppk-tdGFP construct and visualized in fixed dissected brains by immunostaining with mouse anti-GFP primary antibody (DSHB-GFP-8H11) at 1:100 dilution and goat antimouse Alexa488-conjugated secondary antibody (Invitrogen) at 1:250 dilution. Fluorescent images were acquired by Zeiss Axio Imager microscope and analyzed using Zeiss Zen and ImageJ (NIH) software.

### In-solution tryptic digestion, deglycosylation, and LC–MS/MS analyses

Purified protein samples were eluted from anti-FLAG M2 beads (Sigma) with glycine (pH 2.5) and transferred into Tris–HCl. Eluted protein was then reduced, alkylated, and digested with sequence-grade trypsin (Promega). The resulting peptides were desalted and concentrated utilizing microspin C18 columns (The NEST Group) and dried using a vacuum centrifuge. Tryptic peptides were sequentially N-linked deglycosylated with PNGase F and PNGase A. An additional C18 clean-up was performed following deglycosylation. Peptides were separated on a 15 cm C18 analytical PepMap Column (Thermo Fisher Scientific) and eluted into an Orbitrap Fusion Tribrid mass spectrometer (Thermo Fisher Scientific) utilizing a nano–electrospray ionization source *via* a 180 min gradient of increasing buffer B (80% acetonitrile in 0.1% formic acid) at a flow rate of approximately 200 nl/min. Full MS scans were acquired every 3 s by collecting ions between 100 and 1900 *m/z* for a maximum of 100 ms and scanning them in the Orbitrap at 60 K resolution. Following each full scan, the most intense parent ions were subjected to filters before being selected for MS–MS analysis until the next full scan. Every parent ion that met monoisotopic precursor expectations for a peptide, had a charge state of at least 2+, was above 1.0e3 signal, and was not excluded by dynamic exclusion settings was selected for fragmentation. Each selected parent ion was isolated by the quadrupole with an isolation window of 2.0 *m/z*. Each isolated packet was subjected to sHCD before being scanned out in the Orbitrap. Each sHCD spectra contains fragment ions collected from 15%, 25%, and 35% HCD activation. Each parent ion packet was also subjected to CID activation and scanned out from the ion trap. The CID spectra were searched on the fly for the neutral loss of hexose from the parent mass. When this loss was detected, an additional MS3 level scan was performed on the neutral loss peak observed in MS2. Each MS3 event was collected utilizing CID. Any parent ion selected a second time within 10 s was excluded from the selection process for the subsequent 20 s by using the dynamic exclusion node. Blank injections of buffer A (0.1% formic acid) were performed in between each sample, utilizing the same gradient, to limit the possibility of sample carryover.

Raw files were searched utilizing Proteome Discoverer’s Sequest HT (Thermo Fisher Scientific) and Byonic (Protein Metrics) search algorithms against a protein database containing common contaminants and the target protein PTP69D (*Drosophila melanogaster*) (Byonic v4.1.10, PD 2.5.0.400). Search settings were as follows: strict tryptic enzymatic cleavages allowing for up to two missed cleavages, precursor tolerance of 20 ppm, fragment ion tolerance of 20 ppm, fixed carbamidomethylation of cysteine (+57 Da), variable oxidation of methionine (+16 Da), variable deamidation of asparagine (+0.98 Da), variable hexose-modified serine/threonine (+162.05 Da), and variable HexNAc-modified asparagine/serine/threonine (+203.08 Da). Glycopeptides were confirmed by accurate mass (≤2 ppm), false discovery rate less than 5%, and manually confirmed for neutral loss in MS2 (neutral loss peak top peak) and fragmentation in MS3 manually confirmed.

### Analysis of O-hexose by POMGnT1 treatment followed by mass spectrometry

Additional sample of purified protein from POMT1 overexpression was digested utilizing an S-Trap column (Protifi). In this instance, protein was reduced, alkylated, trapped on-column, digested overnight on-column, and eluted off S-Trap the following morning. The resulting peptides were desalted and concentrated utilizing microspin C18 columns and dried using a vacuum centrifuge. Tryptic peptides were sequentially deglycosylated with PNGase F and PNGase A. An additional C18 clean-up was performed following deglycosylation. This sample was then treated with recombinant POMGnT1 in the presence of UDP-GlcNAc to extend any existing O-mannose glycopeptides with a GlcNAc. The reaction with POMGnT1 was carried out overnight at 37 ^°^C in 50 mM sodium acetate buffer including 50 mM UDP-GlcNAc and 50 mM manganese chloride, pH 5.5. The reaction was acidified with formic acid, and the resulting peptides were desalted *via* C18 and dried. After a final C18 clean-up step, these peptides were shot using the same instrument method described previously. Database searching was altered to include fixed methylthiol (+45.98 Da) of cysteine, variable oxidation of methionine (+16 Da), variable deamidation of asparagine (+0.98 Da), variable hexose-modified serine/threonine (+162.05 Da), and variable Hex-HexNAc-modified serine/threonine (+365.13 Da). Glycopeptides were confirmed by accurate mass (≤2 ppm), false discovery rate less than 5%, the presence of signature HexNAc oxonium ions, and the presence of key b and y ions with and without loss of modification.

## Data availability

Data described in the article are shown in the figures. The mass spectrometry proteomics data have been deposited to the ProteomeXchange Consortium *via* the PRIDE partner repository (65) with the dataset identifier PXD034563.

## Supporting information

This article contains supporting information.

## Conflict of interest

The authors declare that they have no conflicts of interest with the contents of this article.
