# Supplementary material for: Protein tyrosine phosphatase 69D is a substrate of protein O-mannosyltransferases 1-2 that is required for the wiring of sensory axons in Drosophila
Source: J Biol Chem. 2023 Jan 10;299(3):102890. doi: 10.1016/j.jbc.2023.102890 (PMC9950532; doi:10.1016/j.jbc.2023.102890)

Receptor Protein Tyrosine Phosphatase 69D is a functional substrate of Protein O-Mannosyltransferases 1-2 required for the wiring of sensory axons in *Drosophila*

Pedro Monagas-Valentin[1], Robert Bridger[2], Ishita Chandel[1], Melissa Koff[1], Boris Novikov[1], Patrick Schroeder[1], Lance Wells[2], and Vladislav Panin[1].
[1] Department of Biochemistry and Biophysics, AgriLife Research, Texas A&M University, College Station, TX 77843, USA; [2] Complex Carbohydrate Research Center, University of Georgia, Athens, GA 30602, USA,

Supplementary Materials

Supplementary Experimental Procedures

Design and cloning of UAS-PTP69D-EC-FLAG

The coding sequence of the extracellular part of PTP69D was PCR-amplifies from full-length *PTP69D* cDNA clone (Drosophila Gene Collection Gold, clone *RE06719*, Berkeley Drosophila Genome Project) using primers *Ptp69d-frw* and *Ptp69D-rev* (Table S1). The PCR product (~ 4.5 kb) was digested with Kpn I and Bam HI restrictases and cloned into similarly digested pBluescript II plasmid, which produced an intermediate construct *pBluescript-PTP69D-EC.* 3FLAG-coding region was introduced into *pBluescript-PTP69D-EC* by cloning a double-stranded oligo encoding 3FLAG peptide between Bam HI and Eag I sites. The double-stranded oligo was generated by synthesizing and annealing two single-stranded oligonucleotides, *3FLAG-top* and *3FLAG-btm* (Table S1). The resulting *pBluescript-PTP69D-EC-FLAG* construct was used as a template to PCR-amplify the sequence encoding PTP69D-EC-FLAG with *Ptp69d-frw2* and *Ptp69d-rev2* primers (Table S1). This PCR product was digested with Kpn I and Xba I restrictases and cloned into similarly prepared *pUAST* plasmid, to produce *UAS-PTP69D-EC-FLAG.* The final construct was confirmed by sequencing.

Table S1. Oligonucleotides used in molecular cloning procedures

**Primer / Oligo**	**Sequence** (*from 3' to 5'*)
Ptp69d-frw1	TGGGTACCAACATGGCGTTACTCTACCGACGCATG
Ptp69D-rev1	ATCGGATCCGGGCAGTTCAGAATCGGAATTTG
3FLAG-top	GATCCGATTACAAAGACCATGACGGTGATTATAAAGATCATGACATCGA TTACAAGGATGACGATGACAAGTAAATCTAGAGC
3FLAG-btm	GGCCGCTCTAGATTTACTTGTCATCGTCATCCTTGTAATCGATGTCATGA TCTTTATAATCACCGTCATGGTCTTTGTAATCG
Ptp69d-frw2	ATAGGTACCAAAATGGCGTTACTCTAC
Ptp69D-rev2	CCGCTCTAGATTTACTTGTCATC

Supplementary Figures

Supplementary Figure S1. Identification of N-linked glycan site modifications of PTP69D-EC-FLAG. Treatment of N-glycosylated glycopeptide with PNGase F / PNGase A cleaves N-linked glycans while creating deamidated asparagine residues, which results in N to D conversion at the sites of modification. Panels show MS spectra of PNGase F / PNGase A treated glycopeptides:

A, N(40)VSLECASENEAVAWK; **B**, LGN(58)QTINK; **C**, SNDDGSENN(85)DSQDFIK; **D**, IYLN(255)WTVNDGNDPIQK; **E**,TCGPWSENVN(429)GTTMDGVATK; **F**, PTNLSIQCHHD**N**(451)VTR; **G,** LNIATYQEVHSDN(613)VTR (MS2 and MS3 spectra)

A

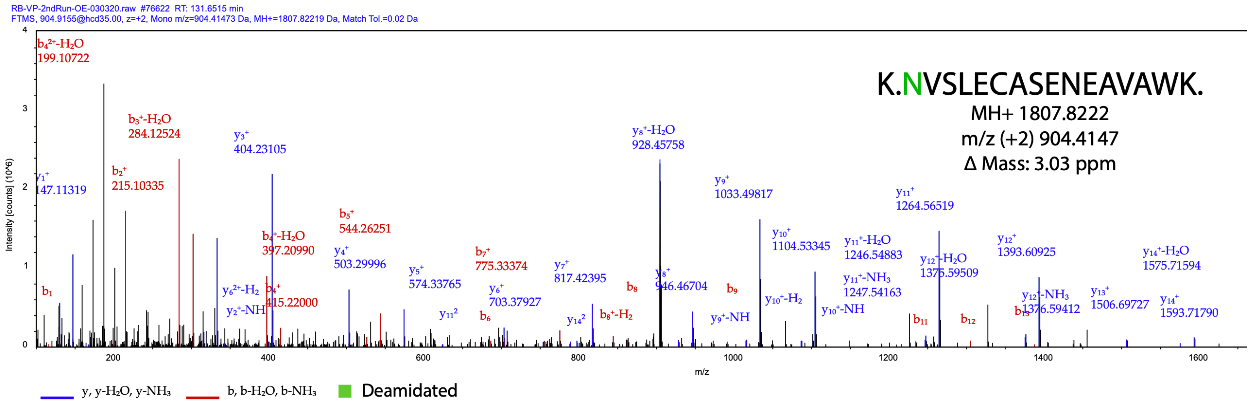

B

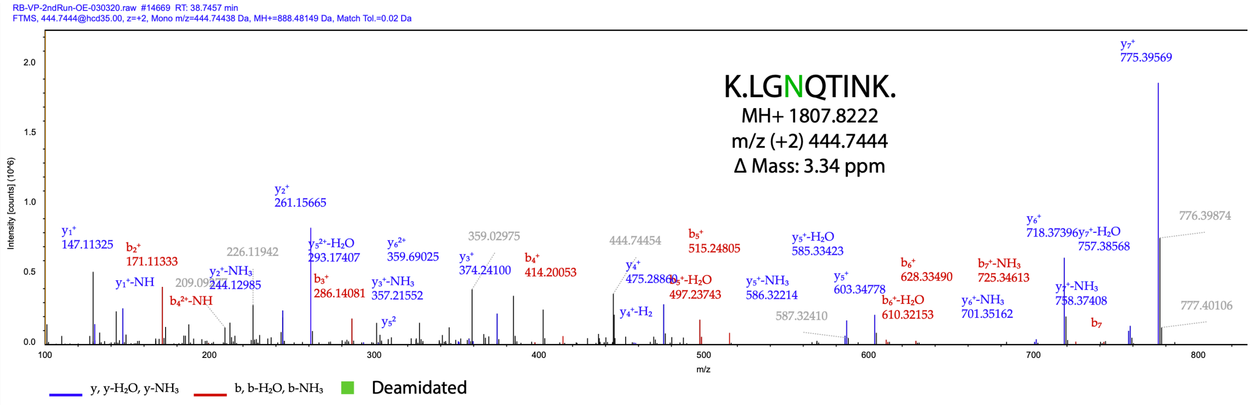

C

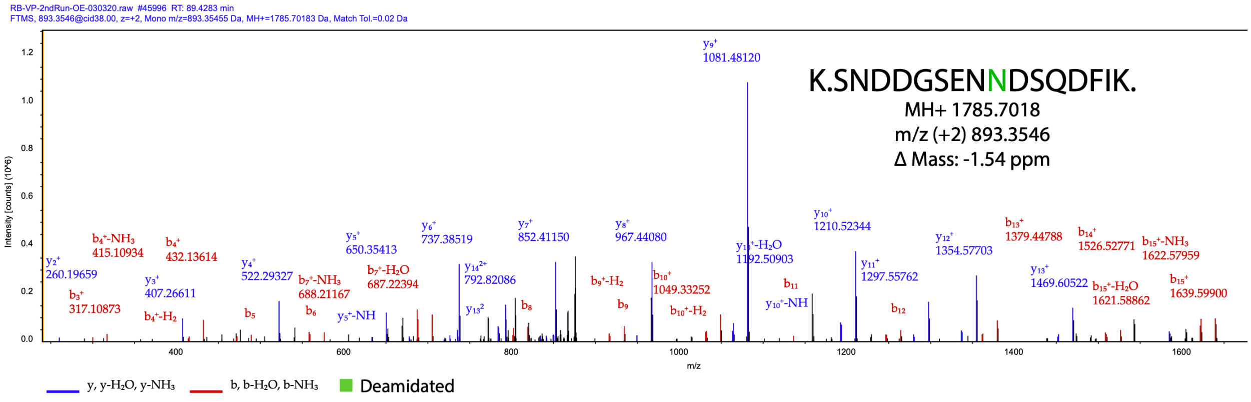

D

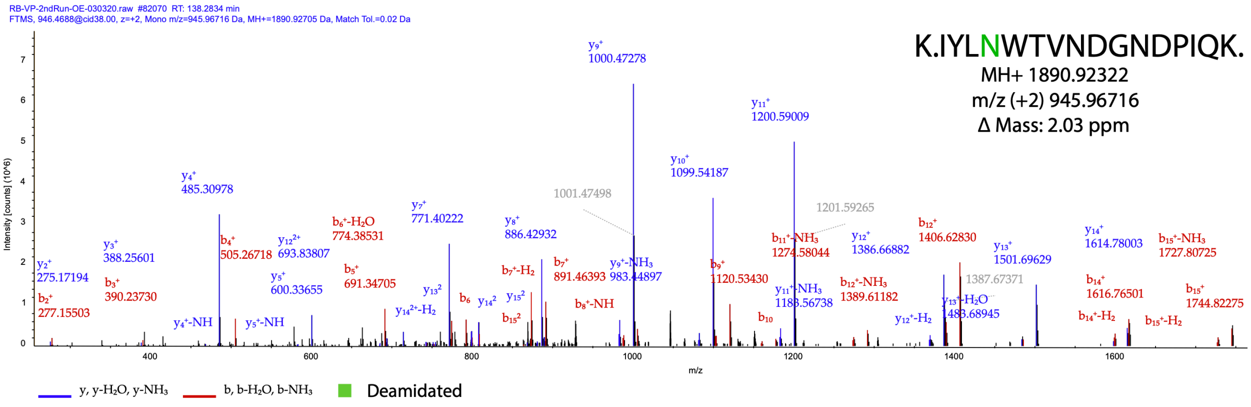
RB-VP-2ndRun-OE-030320.raw #82070 RT: 138.2834 min
FTMS, 946.4688@cid38.00, z=+2, Mono m/z=945.96716 Da, MH+=1890.92705 Da, Match Tol.=0.02 Da
K.IYLNWTVNDGNDPIQK.
MH+ 1890.92322
m/z (+2) 945.96716
Δ Mass: 2.03 ppm
Intensity [counts] (10^6)
m/z
y, y-H2O, y-NH3
b, b-H2O, b-NH3
Deamidated

E

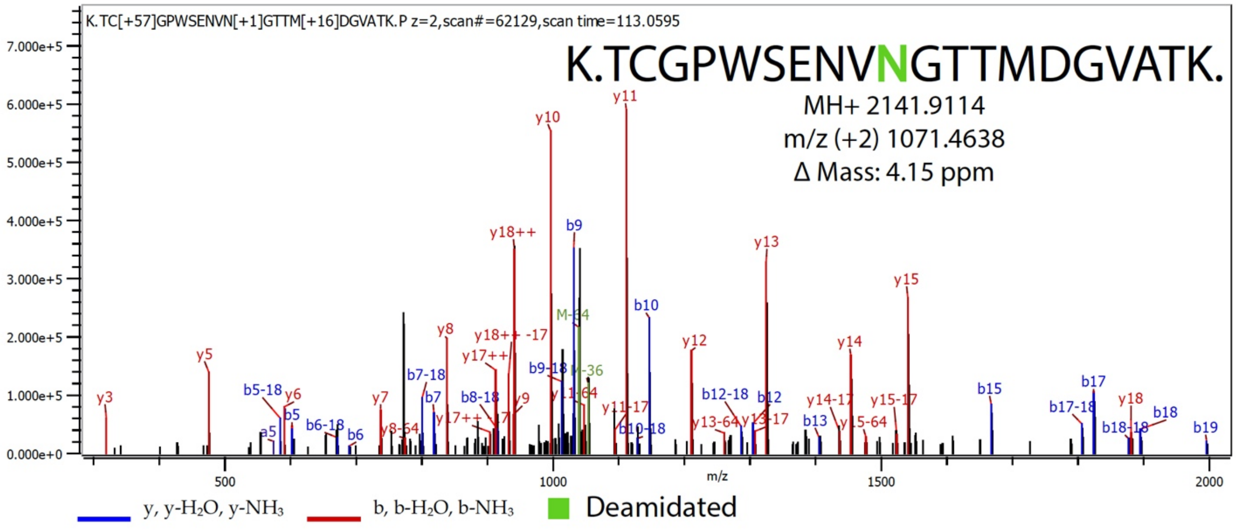
K.TC[+57]GPWSENVN[+1]GTTM[+16]DGVATK.P z=2,scan#=62129,scan time=113.0595
K.TCGPWSENVNGTTMDGVATK.
MH+ 2141.9114
m/z (+2) 1071.4638
Δ Mass: 4.15 ppm
m/z
y, y-H2O, y-NH3
b, b-H2O, b-NH3
Deamidated

F

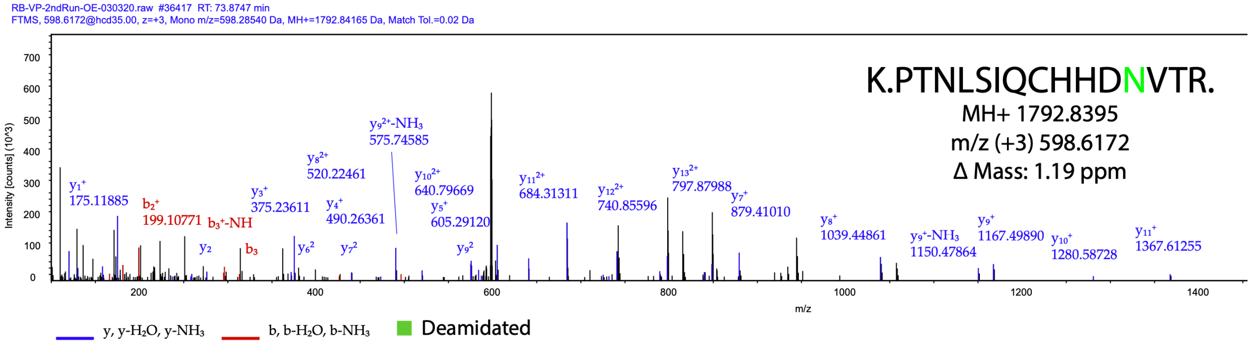
RB-VP-2ndRun-OE-030320.raw #36417 RT: 73.8747 min
FTMS, 598.6172@hcd35.00, z=+3, Mono m/z=598.28540 Da, MH+=1792.84165 Da, Match Tol.=0.02 Da
K.PTNLSIQCHHDNVTR.
MH+ 1792.8395
m/z (+3) 598.6172
Δ Mass: 1.19 ppm
Intensity [counts] (10^3)
m/z
y, y-H2O, y-NH3
b, b-H2O, b-NH3
Deamidated

G

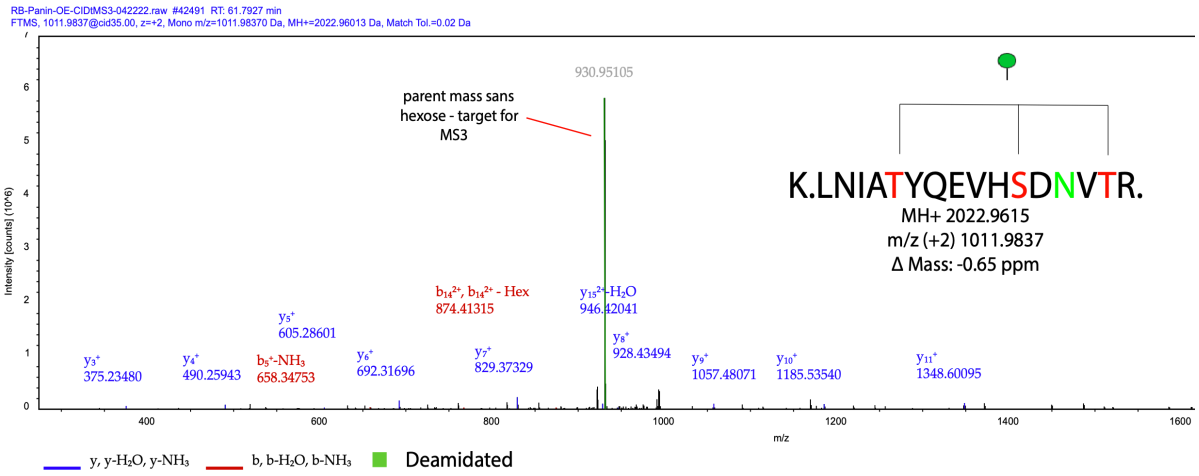
RB-Panin-OE-CIDtMS3-042222.raw #42491 RT: 61.7927 min
FTMS, 1011.9837@cid35.00, z=+2, Mono m/z=1011.98370 Da, MH+=2022.96013 Da, Match Tol.=0.02 Da
930.95105
parent mass sans hexose - target for MS3
K.LNIATYQEVHSDNVTR.
MH+ 2022.9615
m/z (+2) 1011.9837
Δ Mass: -0.65 ppm
Intensity [counts] (10^6)
m/z
y, y-H2O, y-NH3
b, b-H2O, b-NH3
Deamidated

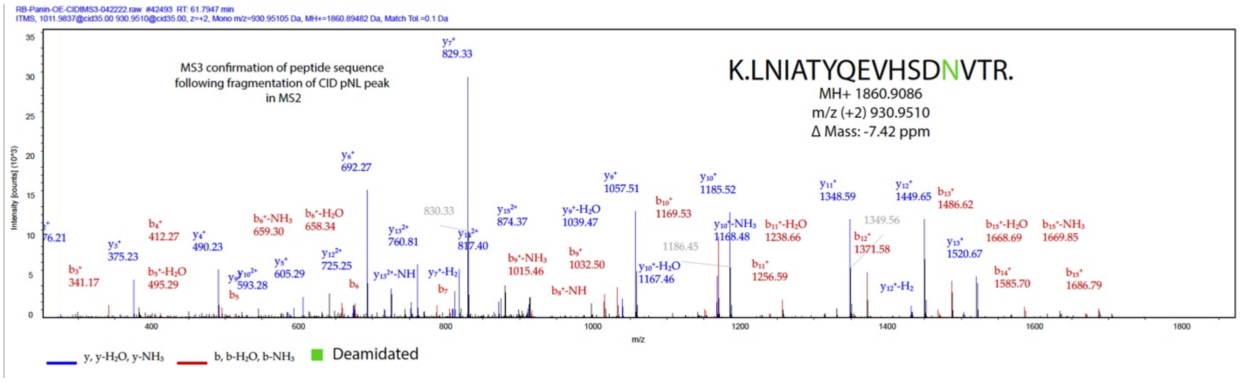
RB-Panin-OE-CIDtMS3-042222.raw #42493 RT: 61.7947 min
ITMS, 1011.9837@cid35.00 930.9510@cid35.00, z=+2, Mono m/z=930.95105 Da, MH+=1860.89482 Da, Match Tol.=0.1 Da
MS3 confirmation of peptide sequence following fragmentation of CID pNL peak in MS2
K.LNIATYQEVHSDNVTR.
MH+ 1860.9086
m/z (+2) 930.9510
Δ Mass: -7.42 ppm
Intensity [counts] (10^3)
m/z
y, y-H2O, y-NH3
b, b-H2O, b-NH3
Deamidated

Supplementary Figure 2. Identification of O-linked mannose site modifications of PTP69D-EC-FLAG using CID neutral loss and sHCD spectral analysis. **A,** LGNQT(60)INK; **B**, T(72)EPLK; **C**, VIEEAIYQQNS(391)R, MS2 and MS3 spectra; **D**, LNIAT(605)YQEVHS(611)DNVT(615)R, one hexose is attached but the site was not unambiguously determined; **E**, SS(617/618)AYIAEMISS(626/627), one hexose is attached but the site was not unambiguously determined, MS2 and MS3 spectra; **F**, SS(617/618)AYIAEMISS(626/627), three hexoses are attached but the sites were not unambiguously determined, MS2 and MS3 spectra; **G**, SS(617/618)AYIAEMISS(626/627), four hexoses are attached, MS2 spectrum.

A

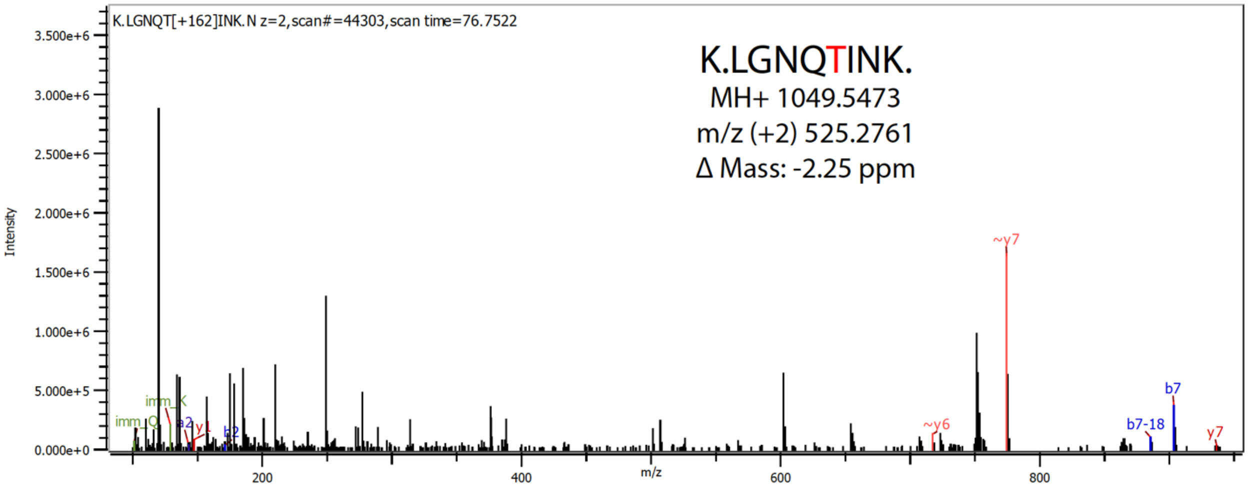

B

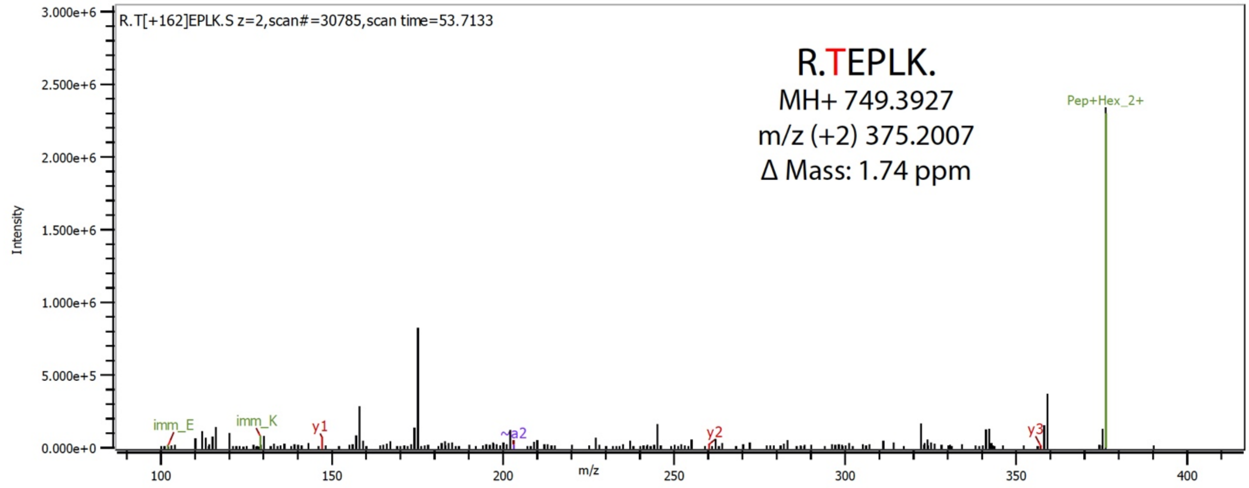

C

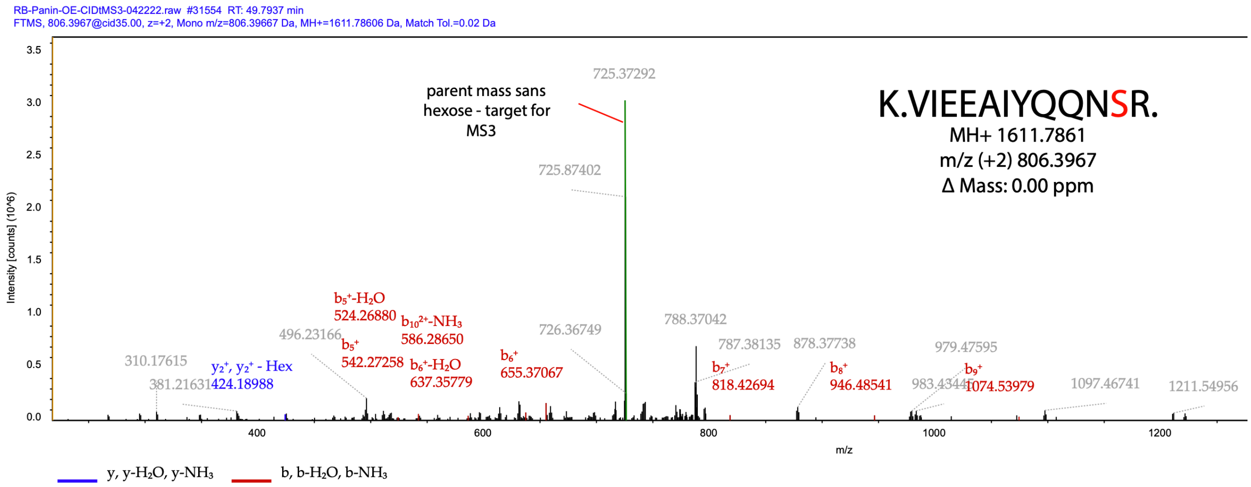

RB-Panin-OE-CIDtMS3-042222.raw #31554 RT: 49.7937 min
FTMS, 806.3967@cid35.00, z=+2, Mono m/z=806.39667 Da, MH+=1611.78606 Da, Match Tol.=0.02 Da
parent mass sans hexose - target for MS3
K.VIEEAIYQQNSR.
MH+ 1611.7861
m/z (+2) 806.3967
Δ Mass: 0.00 ppm
Intensity [counts] (10^6)
m/z
y, y-H2O, y-NH3
b, b-H2O, b-NH3

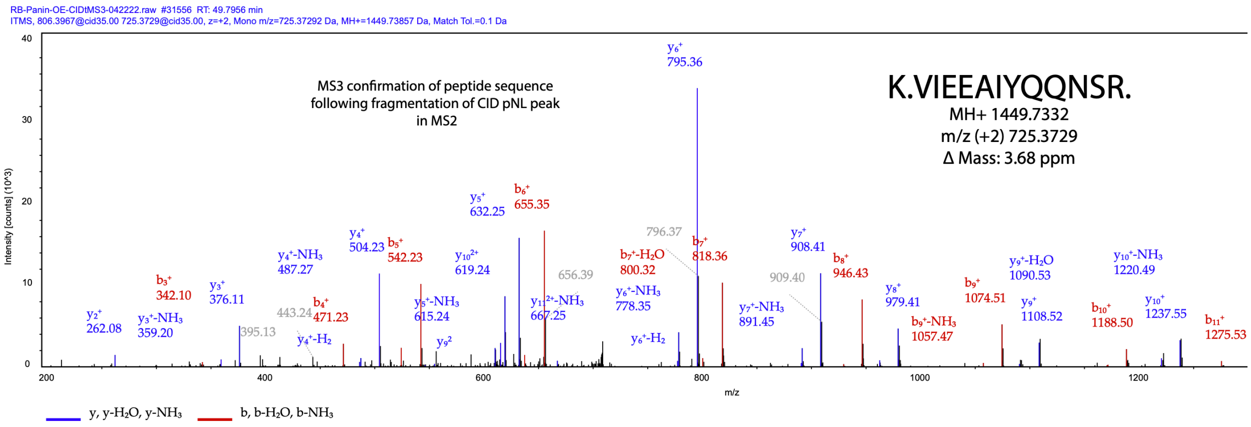

RB-Panin-OE-CIDtMS3-042222.raw #31556 RT: 49.7956 min
ITMS, 806.3967@cid35.00 725.3729@cid35.00, z=+2, Mono m/z=725.37292 Da, MH+=1449.73857 Da, Match Tol.=0.1 Da
MS3 confirmation of peptide sequence following fragmentation of CID pNL peak in MS2
K.VIEEAIYQQNSR.
MH+ 1449.7332
m/z (+2) 725.3729
Δ Mass: 3.68 ppm
Intensity [counts] (10^3)
m/z
y, y-H2O, y-NH3
b, b-H2O, b-NH3

D

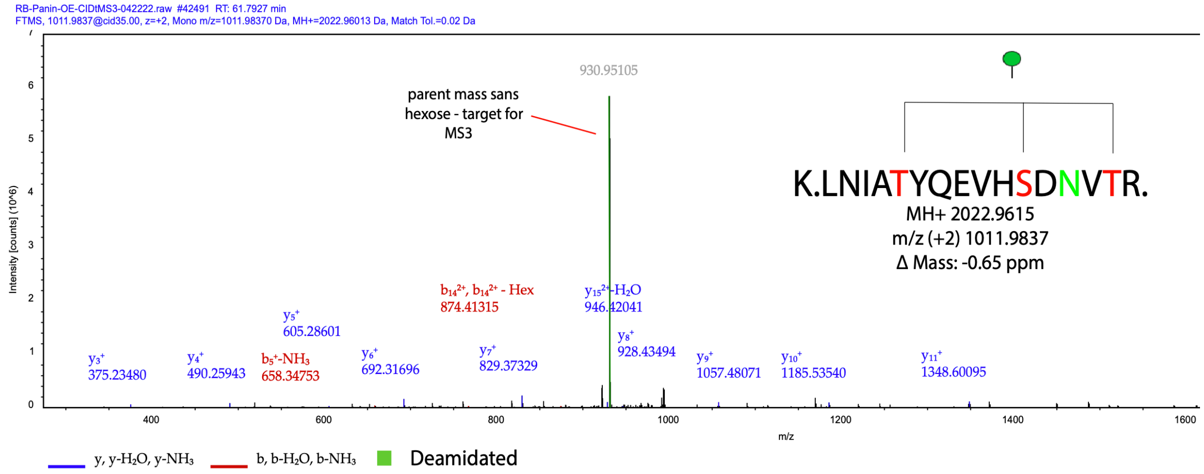
RB-Panin-OE-CIDtMS3-042222.raw #42491 RT: 61.7927 min
FTMS, 1011.9837@cid35.00, z=+2, Mono m/z=1011.98370 Da, MH+=2022.96013 Da, Match Tol.=0.02 Da
930.95105
parent mass sans hexose - target for MS3
K.LNIATYQEVHSDNVTR.
MH+ 2022.9615
m/z (+2) 1011.9837
Δ Mass: -0.65 ppm
b14 2+, b14 2+ - Hex 874.41315
y15 2+-H2O 946.42041
y5+ 605.28601
y8+ 928.43494
y3+ 375.23480
y4+ 490.25943
b5+-NH3 658.34753
y6+ 692.31696
y7+ 829.37329
y9+ 1057.48071
y10+ 1185.53540
y11+ 1348.60095
Intensity [counts] (10^6)
m/z
y, y-H2O, y-NH3
b, b-H2O, b-NH3
Deamidated

E

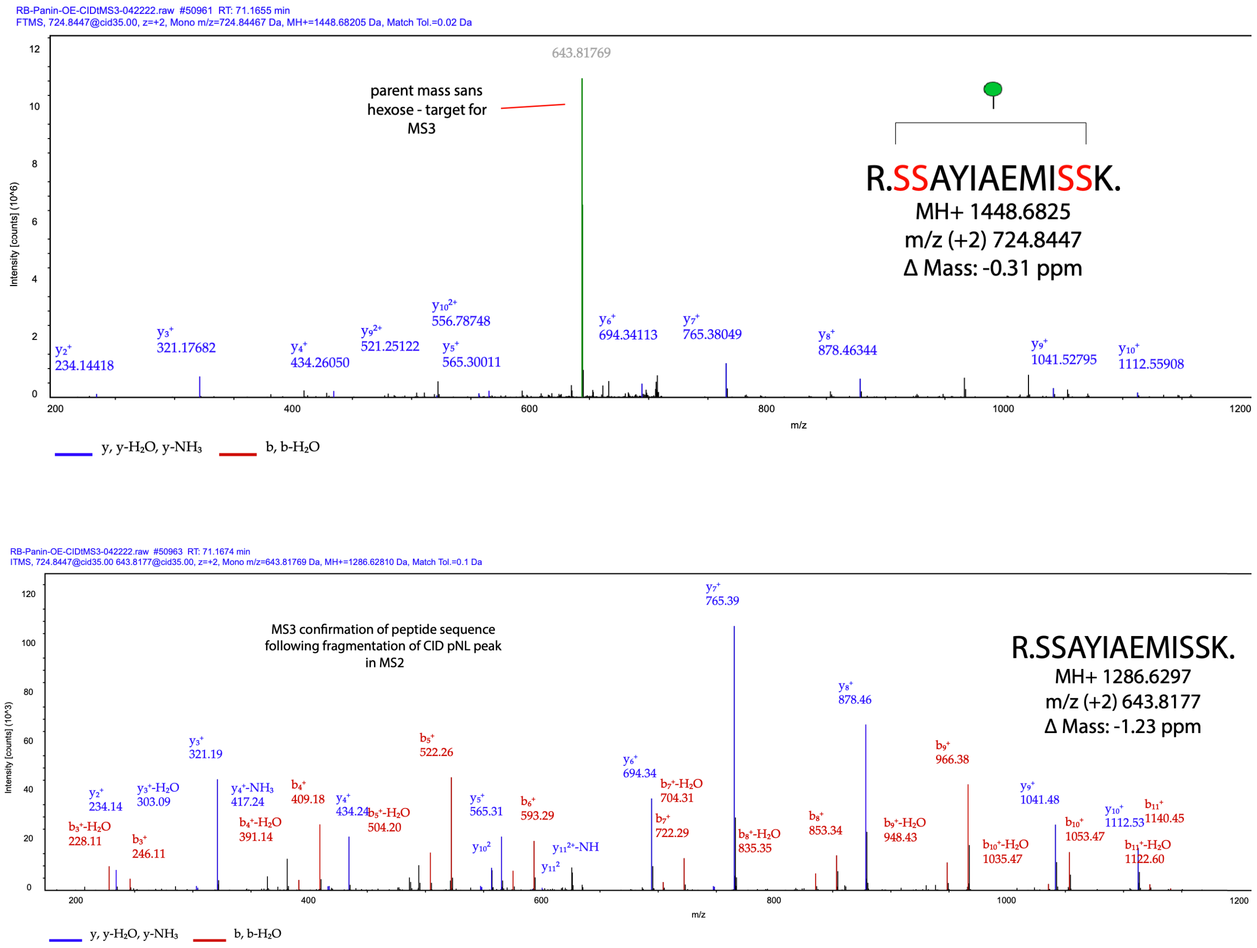
RB-Panin-OE-CIDtMS3-042222.raw #50961 RT: 71.1655 min
FTMS, 724.8447@cid35.00, z=+2, Mono m/z=724.84467 Da, MH+=1448.68205 Da, Match Tol.=0.02 Da
643.81769
parent mass sans hexose - target for MS3
R.SSAYIAEMISSK.
MH+ 1448.6825
m/z (+2) 724.8447
Δ Mass: -0.31 ppm
y2+ 234.14418
y3+ 321.17682
y4+ 434.26050
y9 2+ 521.25122
y10 2+ 556.78748
y5+ 565.30011
y6+ 694.34113
y7+ 765.38049
y8+ 878.46344
y9+ 1041.52795
y10+ 1112.55908
Intensity [counts] (10^6)
m/z
y, y-H2O, y-NH3
b, b-H2O
RB-Panin-OE-CIDtMS3-042222.raw #50963 RT: 71.1674 min
ITMS, 724.8447@cid35.00 643.8177@cid35.00, z=+2, Mono m/z=643.81769 Da, MH+=1286.62810 Da, Match Tol.=0.1 Da
MS3 confirmation of peptide sequence following fragmentation of CID pNL peak in MS2
R.SSAYIAEMISSK.
MH+ 1286.6297
m/z (+2) 643.8177
Δ Mass: -1.23 ppm
y7+ 765.39
y8+ 878.46
y3+ 321.19
b5+ 522.26
b9+ 966.38
y6+ 694.34
b7+-H2O 704.31
y9+ 1041.48
y2+ 234.14
y3+-H2O 303.09
y4+-NH3 417.24
b4+ 409.18
y4+ 434.24
b5+-H2O 504.20
y5+ 565.31
b6+ 593.29
b7+ 722.29
b8+-H2O 835.35
b8+ 853.34
b9+-H2O 948.43
b10+ 1053.47
y10+ 1112.53
b11+ 1140.45
b3+-H2O 228.11
b3+ 246.11
b4+-H2O 391.14
y10 2
y11 2+-NH
y11 2
b10+-H2O 1035.47
b11+-H2O 1122.60
Intensity [counts] (10^3)
m/z
y, y-H2O, y-NH3
b, b-H2O

F

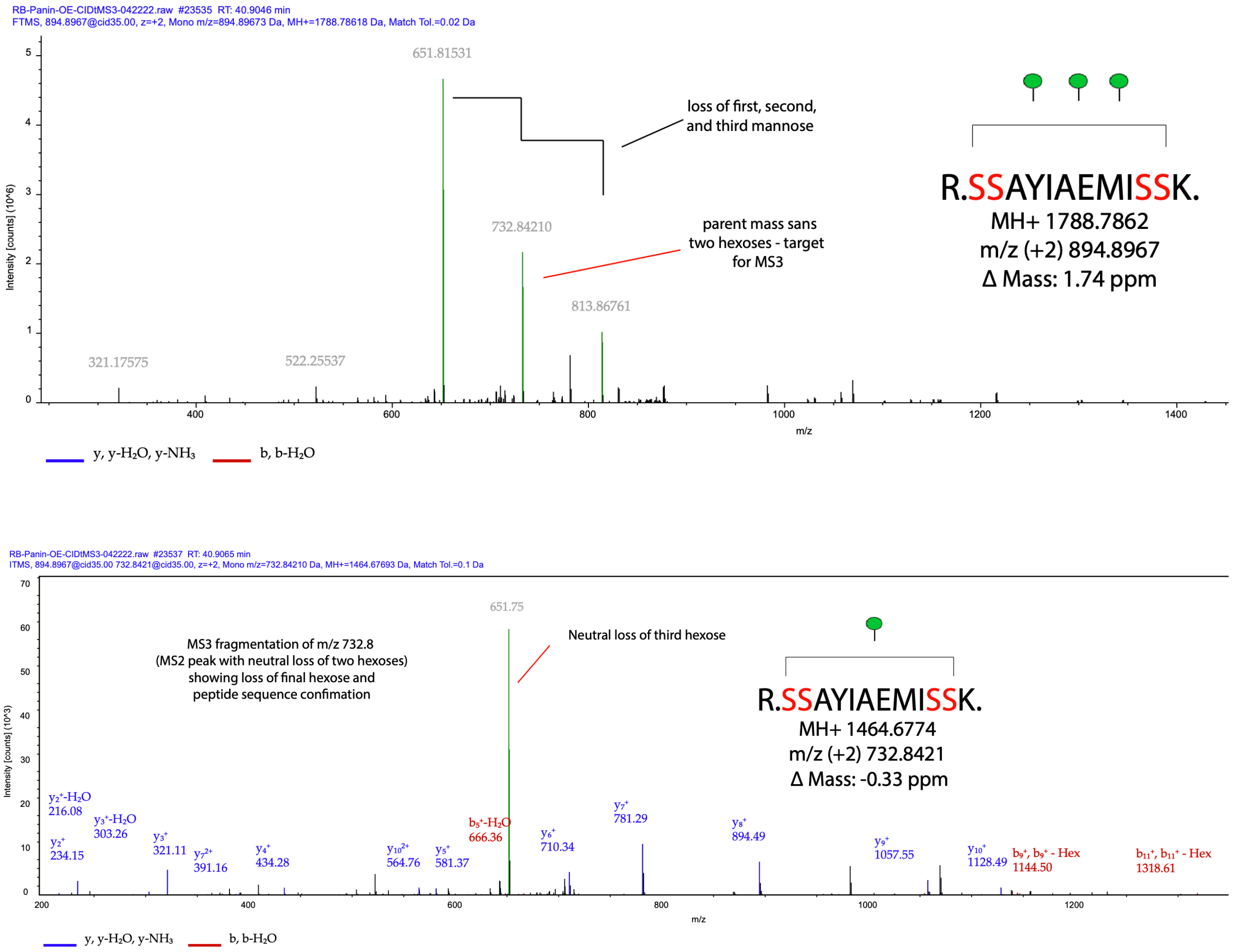
RB-Panin-OE-CIDtMS3-042222.raw #23535 RT: 40.9046 min
FTMS, 894.8967@cid35.00, z=+2, Mono m/z=894.89673 Da, MH+=1788.78618 Da, Match Tol.=0.02 Da
Intensity [counts] (10^6)
651.81531
732.84210
813.86761
321.17575
522.25537
loss of first, second, and third mannose
parent mass sans two hexoses - target for MS3
R.SSAYIAEMISSK.
MH+ 1788.7862
m/z (+2) 894.8967
Δ Mass: 1.74 ppm
m/z
y, y-H2O, y-NH3
b, b-H2O
RB-Panin-OE-CIDtMS3-042222.raw #23537 RT: 40.9065 min
ITMS, 894.8967@cid35.00 732.8421@cid35.00, z=+2, Mono m/z=732.84210 Da, MH+=1464.67693 Da, Match Tol.=0.1 Da
Intensity [counts] (10^3)
651.75
MS3 fragmentation of m/z 732.8
(MS2 peak with neutral loss of two hexoses)
showing loss of final hexose and
peptide sequence confimation
Neutral loss of third hexose
R.SSAYIAEMISSK.
MH+ 1464.6774
m/z (+2) 732.8421
Δ Mass: -0.33 ppm
y2+-H2O 216.08
y2+ 234.15
y3+-H2O 303.26
y3+ 321.11
y7 2+ 391.16
y4+ 434.28
y10 2+ 564.76
y5+ 581.37
b5+-H2O 666.36
y6+ 710.34
y7+ 781.29
y8+ 894.49
y9+ 1057.55
y10+ 1128.49
b9+, b9+ - Hex 1144.50
b11+, b11+ - Hex 1318.61
m/z
y, y-H2O, y-NH3
b, b-H2O

G

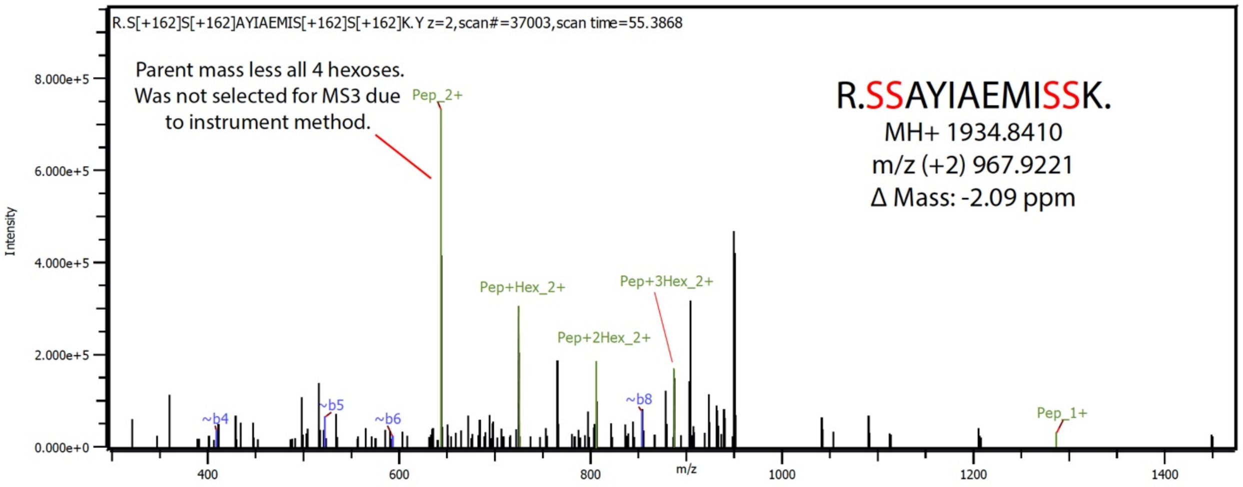
R.S[+162]S[+162]AYIAEMIS[+162]S[+162]K.Y z=2,scan#=37003,scan time=55.3868
Parent mass less all 4 hexoses.
Was not selected for MS3 due
to instrument method.
Pep_2+
Pep+Hex_2+
Pep+2Hex_2+
Pep+3Hex_2+
Pep_1+
~b4
~b5
~b6
~b8
R.SSAYIAEMISSK.
MH+ 1934.8410
m/z (+2) 967.9221
Δ Mass: -2.09 ppm
Intensity
m/z

Supplementary Figure 3. Identification of O-linked mannose modifications of PTP69D-EC-FLAG by applying treatment with POMGnT1 followed by MS analyses. SS(617/618)AYIAEMISS(626/627) was found to be modified with 2 hexoses and a HexNAc-Hex. Positions of modifications were not unambiguously determined.

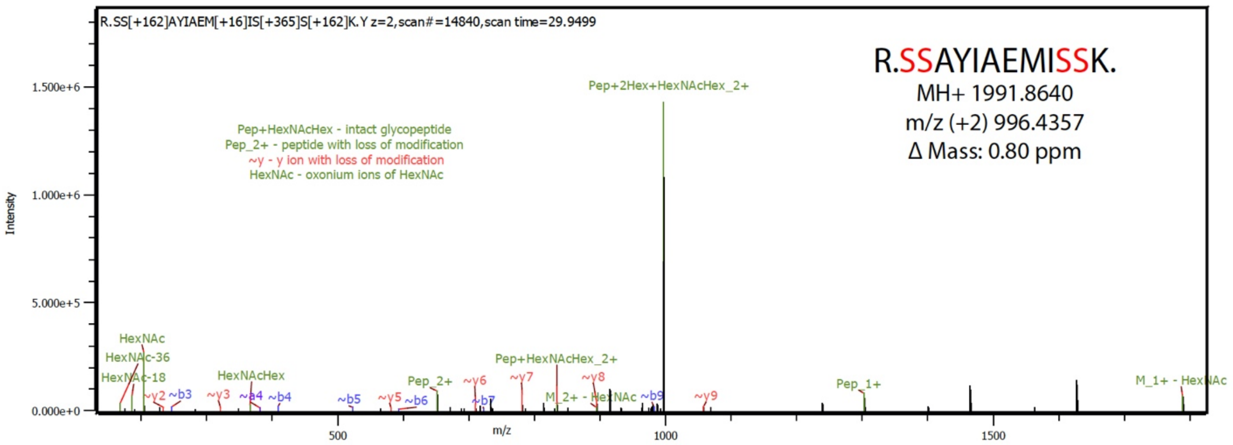

Supplement: Supplementary materials [file mmc1.pdf]
